# A genome-wide association study implicates multiple mechanisms influencing raised urinary albumin–creatinine ratio

**DOI:** 10.1093/hmg/ddz243

**Published:** 2019-10-20

**Authors:** Francesco Casanova, Jessica Tyrrell, Robin N Beaumont, Yingjie Ji, Samuel E Jones, Andrew T Hattersley, Michael N Weedon, Anna Murray, Angela C Shore, Timothy M Frayling, Andrew R Wood

**Affiliations:** 1 Diabetes and Vascular Medicine, NIHR Exeter Clinical Research Facility and College of Medicine and Health, University of Exeter, Exeter, UK; 2 Genetics of Complex Traits, College of Medicine and Health, University of Exeter, Exeter, UK; 3 Institute of Biomedical & Clinical Science, University of Exeter, Exeter, UK

## Abstract

Raised albumin–creatinine ratio (ACR) is an indicator of microvascular damage and renal disease. We aimed to identify genetic variants associated with raised ACR and study the implications of carrying multiple ACR-raising alleles with metabolic and vascular-related disease. We performed a genome-wide association study of ACR using 437 027 individuals from the UK Biobank in the discovery phase, 54 527 more than previous studies, and followed up our findings in independent studies. We identified 62 independent associations with ACR across 56 loci (*P* < 5 × 10^–8^), of which 20 were not previously reported. Pathway analyses and the identification of 20 of the 62 variants (at *r*^2^ > 0.8) coinciding with signals for at least 16 related metabolic and vascular traits, suggested multiple pathways leading to raised ACR levels. After excluding variants at the *CUBN* locus, known to alter ACR via effects on renal absorption, an ACR genetic risk score was associated with a higher risk of hypertension, and less strongly, type 2 diabetes and stroke. For some rare genotype combinations at the *CUBN* locus, most individuals had ACR levels above the microalbuminuria clinical threshold. Contrary to our hypothesis, individuals carrying more *CUBN* ACR-raising alleles, and above the clinical threshold, had a higher frequency of vascular disease. The *CUBN* allele effects on ACR were twice as strong in people with diabetes—a result robust to an optimization-algorithm approach to simulating interactions, validating previously reported gene–diabetes interactions (*P* ≤ 4 × 10^–5^). In conclusion, a variety of genetic mechanisms and traits contribute to variation in ACR.

## Introduction

High urinary albumin excretion is a marker of chronic kidney disease (CKD) and a predictor of mortality and cardiovascular events in the general population and in clinical populations, such as individuals with diabetes (1). Moreover, raised albuminuria is believed to be indicative of systemic endothelial microvascular damage (2). Albumin–creatinine ratio (ACR) is an accepted marker of urinary excretion of albumin (3) available in large numbers of individuals through routine clinical testing.

Genetic studies of ACR are important as individuals with raised ACR levels based on genetics affecting tubular reabsorption, for example, may not be at a higher risk of vascular disease. Conversely, individuals with low ACR levels based on genotypes that directly alter ACR may be missed in clinical tests for microvascular damage. Genetic studies of ACR may identify new, or clarify the role of known, pathways altering microvascular function or kidney function, or both.

Prior to the availability of data from UK Biobank, previous genetic association studies had identified only one region of the genome robustly associated with ACR at the cubilin (*CUBN*) locus (4–6). Associations were mainly represented by a common single-nucleotide polymorphism (SNP) (rs1801239) with a minor allele frequency (MAF) of 10% (5). In addition, van Zuydam *et al.* showed a putative association between a signal on chromosome 6 (*GABRR1*) and diabetic microalbuminuria (7). Most recently, a rare SNP (rs141640975) with a MAF of 0.8% was identified as associated with albuminuria through exome sequencing (8). Ahluwalia *et al.* also identified evidence of association for three genes (*HES1*, *CDC73* and *GRM4*) after performing gene-based tests. Both Teumer *et al.* and Ahluwalia *et al.* provided evidence that the *CUBN* variant has a 3- to 4-fold larger effect in people with diabetes. Teumer *et al.* identified two additional loci with stronger effects in individuals with diabetes (RAB38/*CTSC* and *HS6ST1*).

Mutations in the *CUBN* gene cause an autosomal recessive disorder, Imerslund-Gräsbeck Syndrome, characterized by Vitamin B12 malabsorption and, in many cases, mild proteinuria (9). Proteinuria in this context is likely to be based on a defect of the cubilin receptor which binds albumin in renal tubuli thus decreasing albumin reabsorption (10). Therefore, the *CUBN* variant may alter ACR directly without being associated with renal damage.

To understand more about the genetic factors associated with variation in ACR and study the implications of carrying multiple ACR-raising alleles with metabolic and vascular-related disease, we performed a genome-wide association study (GWAS) of ACR using 437 027 individuals from the UK Biobank study with subsequent replication using publicly available data from the CKDGen consortium (5) and the EXTEND study. Our study follows those from Haas *et al.* and Zanetti *et al.* who identified 46 and 21 genetic associations with ACR at the conventional *P* < 5 × 10^−8^ threshold, respectively, in smaller subsets of UK Biobank participants (*N* = 382 500 and *N* = 218 450 discovery sets, respectively) (11,12). In addition to the identification of 20 novel loci in our larger sample size, we focused our analyses on those very difficult to perform in the context of a GWAS consortium of many smaller studies and not performed by Haas *et al*. These analyses included testing haplotype effects at the *CUBN* locus, colocalization of genetic associations and gene–disease interactions in the UK Biobank.

## Results

Clinical characteristics of the 437 027 UK Biobank individuals of European ancestry and with ACR available are presented in Table 1 and Supplementary Material, Fig. S1. The characteristics of the EXTEND study individuals are also presented inTable 1**.**

**Table 1:** Characteristics of participants from the UK Biobank and EXTEND analyzed. Data presented as mean (±standard deviation) or median [25th–75th percentile] were not otherwise stated

	**UK Biobank**	**EXTEND**
*N*	437 027	5679
Age (years)	57.28 (± 8.02)	54.31 (± 14.83)
Sex (%female—%male)	54.18–45.82	62.85–37.15
Height (cm)	168.7 (± 9.2)	168.5 (± 9.1)
BMI	27.38 (± 4.74)	26.52 (± 4.62)
ACR (mg/mml)	1.08 [0.68–1.82]	0.73 [0.42–1.34]
CAD (%yes—%no)	10.47–89.53	3.79–96.21
T2D (%yes—%no)	3.17–96.83	1.04–98.96
Systolic BP (mmHg)	144.2 (± 24.0)	131.1 (± 19.8)
Diastolic BP (mmHg)	86.3 (± 13.5)	77.4 (± 10.8)

ACR = albumin–creatinine ratio; CAD = coronary artery disease; T2D = type 2 diabetes; BP = blood pressure; BMI = body mass index.

### GWAS of ACR in UK Biobank identified 62 associated variants across 56 loci, 20 of which have not been previously associated with ACR

We identified 62 statistically independent SNPs in 56 loci associated with ACR at *P* < 5 × 10^−8^ of which 42 reached *P* < 6 × 10^−9^, a threshold that we estimate reflects better the 5% type 1 error rate (13). Of the 62 associations, 18 were located in loci not previously reported to be associated with ACR, and 2 were in low linkage disequilibrium (*r*^2^ < 0.1) with lead SNPs previously reported. Of these 20 associations, 9 reached *P* < 6 × 10^−9^ (Table 2). The LD score regression intercept was 1.05 indicating limited inflation based on population stratification. Conditional analysis revealed five loci containing one additional signal and one containing two additional signals (*CUBN*) (Supplementary Material, Table S1 and Figs S2 and S3).

**Table 2:** GWAS summary statistics in the UK Biobank for the 20 SNPs located in loci not previously reported or in low linkage disequilibrium (*r*^2^ < 0.1) with lead SNPs previously reported. Comparisons with publicly available data from the CKDGen consortium and lookups in the EXTEND study can be found in Supplementary Material, Tables S2 and S3, respectively

**Nearest gene**	**SNP**	**Chr**	**Position (b37)**	**EA/OA**	**EA Freq**	**Beta**	**SE**	***P***
*MST01*	rs35202981	1	155578042	G/A	0.139	0.017	0.003	1.4E-08
*EDEM3*	rs78444298	1	184672098	G/A	0.980	0.045	0.007	1.4E-09
*GPD2*	rs111688960	2	157599687	A/G	0.013	0.051	0.009	1.2E-08
*FAT1*	rs62342738	4	187656129	C/G	0.181	0.015	0.003	5.5E-09
*C5orf56*	rs11242113	5	131777234	A/G	0.188	0.016	0.003	1.6E-09
*KCNK5*	rs1544935	6	39124448	G/T	0.216	0.017	0.002	2.2E-11
*VEGFA*	rs3734692	6	43817791	T/A	0.310	0.016	0.002	1.8E-13
*AUTS2*	rs35692677	7	69902654	G/A	0.813	0.015	0.003	1.1E-08
*ZBTB1*	rs11990607	8	81363534	A/G	0.835	0.015	0.003	2.5E-08
*MLLT10*	rs6482189	10	21889138	G/A	0.683	0.013	0.002	1.6E-09
*CYP26A1*	rs2068888	10	94839642	G/A	0.550	0.012	0.002	1.0E-09
*SBF2*	rs11042685	11	10262551	C/T	0.493	0.011	0.002	2.1E-08
*NUMA1*	rs7115200	11	71752160	G/T	0.440	0.012	0.002	1.4E-09
*OAF*	rs12790943	11	120058623	T/C	0.421	0.011	0.002	3.0E-08
*NAV3*	rs10860332	12	78748014	A/G	0.414	0.011	0.002	4.4E-08
*DLEU1/BCMS*	rs3116613	13	51143055	G/T	0.211	0.014	0.002	3.9E-08
*WDR81*	rs550628400	17	1639795	G/A	0.006	0.075	0.013	2.1E-08
*CYP2A7*	rs79600176	19	41392490	T/C	0.978	0.038	0.007	3.6E-08
*CCDC97*	rs56254331	19	41826020	A/C	0.831	0.017	0.003	7.9E-10
*ZBTB46*	rs11697610	20	62379531	G/A	0.387	0.012	0.002	1.9E-08

SNP = single-nucleotide polymorphism; b37 = build 37; EA/OA = effect allele/other allele; Freq = frequency; SE = standard error; *P* = *P* value.

### Genetic variants validate in independent data

We used summary statistics from the CKDGen consortium meta-analysis of 54 451 individuals (5) to check for directional consistency of the ACR-associated variants. Of the 62 variants, 47 were available in the summary statistics (including proxy variants at *r*^2^ > 0.8). The effect estimates of 39/47 SNPs showed directional consistency (*P*_binomial_ < 0.0001) (Supplementary Material, Table S2). The variants not previously identified also showed strong evidence of validation—15 of the 20 were present in the CKDGen consortium (or their proxies), and 11 of these were directionally consistent. In addition, 8/15 analyzed by the CKDGen consortium reaching *P* < 6 × 10^−9^ in our analysis, 7/8 effect estimates were directionally consistent. Of the 47 SNPs, the maximum variance explained by a single SNP in UK Biobank was 0.03%. We note the power to detect an association at *P* = 5 × 10^−8^ explaining 0.03% of the variance in 54.451 individuals was 8%, and 76% at *P* = 8 × 10^−4^ after Bonferroni correction for 62 tests (0.05/62). In the EXTEND study of 5679 individuals with measures of ACR, the overall variance explained by all 62 ACR-associated SNPs was 0.61% of the inverse-normalized trait (Supplementary Material, Table S3).

### High genetic risk for ACR increases risk of microalbuminuria

We next assessed the risk of being above the clinical threshold of 3 mg/mmol for microalbuminuria using a genetic risk score (GRS) for ACR. A one-unit increase in the ACR GRS was equivalent to a 0.07-mg/mmol increase in ACR. In the UK Biobank, a one-unit increase in the GRS for ACR was associated with a higher risk for being over the clinical threshold and this effect was stronger in men (odds ratio (OR) = 1.124, 95% CI: 1.116–1.133, *P* = 4.0 × 10^−202^) than women (OR = 1.059, 95% CI: 1.053–1.065, *P* = 3.4 × 10^−85^; *P*_interaction_ = 9.3 × 10^−34^). To avoid inflated effect estimates due to ‘winner’s curse’, we repeated this analysis in the independent EXTEND study. A one-unit higher GRS for ACR was associated with an overall OR = 1.062 (95% CI: 1.015–1.110, *P* = 0.009). When restricting the analysis to *CUBN-*raising alleles, we showed that the 19.9% of UK Biobank individuals carrying at least one *CUBN* raising allele had an OR = 1.153 (95% CI: 1.122–1.185, *P* = 1.0 × 10^−24^) for being above the clinical threshold in contrast to those carrying no ACR-raising alleles at the locus.

### Individuals with a GRS for high ACR have a higher risk of hypertension

We next tested the combined role of ACR-associated variants in five diseases related to vascular dysfunction in the UK Biobank: hypertension, type 2 diabetes (T2D), coronary artery disease (CAD), CKD and stroke. We reasoned that this would reveal whether individuals with a high ACR GRS were at high risk of vascular-related disease. The GRS of raised ACR was associated with higher risk of hypertension [OR = 1.013, 95% CI: 1.010–1.016, *P* = 1.6 × 10^−16^], but much weaker or no evidence of association with stroke [OR = 1.011, 95% CI: 1.001–1.022, *P* = 0.027], T2D [OR = 1.008, 95% CI: 1.000–1.017, *P* = 0.045], CAD [OR = 1.004, 95% CI: 0.999–1.010, *P* = 0.13] and CKD [OR = 0.996, 95% CI: 0.982–1.010, *P* = 0.59]. These results were not strongly influenced by the large effect of SNPs in *CUBN*—we obtained similar results when using a GRS excluding the three variants in *CUBN* (Supplementary Material, Table S4). We had no direct measure of vascular disease, and therefore, we were unable to establish if the association between the ACR GRS and hypertension was directly based on vascular dysfunction, but they imply that a higher GRS for ACR is not benign.

### Analyses of pathways and variants previously associated with vascular and metabolic traits implicate multiple mechanisms

We next examined the 62 ACR-associated variants for pleiotropic effects. We observed these variants to likely influence ACR through a wide variety of mechanisms, many of which are known to be causally related to, or strongly associated with, vascular diseases and related traits. Previous GWAS had identified 20 of the ACR-associated variants (or those in strong LD (*r*^2^ > 0.8)) as associated with a trait related to metabolic, inflammatory or vascular disease at genome-wide significance levels (*P* < 5 × 10^−8^). These associations were with a wide variety of traits, including blood lipid profiles, fasting glucose, blood pressure and T2D (Supplementary Material, Table S5). Two variants represented known T2D signals (a variant in the *ARL15* locus and a variant in the *SNX17* locus that is in LD with highly pleiotropic variants at the *GCKR* locus (*r^2^* > 0.85)), three represented known CAD signals (a variant near *KCKN5*, one near *TRIB1*, and one in the intron of *CCDC97*) and one represented a known blood pressure signal (a variant in *HOTTIP*). These variants are likely examples of variants with pleiotropic effects that affect ACR through additional mechanisms. For 7 of the 20 ACR-associated variants in strong linkage disequilibrium with known signals for other traits, data was available to perform a colocalization analysis. All seven showed a high probability (>0.7) that variants associated with ACR represented the same signal as that previously reported—including with those for blood lipid profiles, fasting glucose, blood pressure and T2D (Supplementary Material, Table S6). Using MAGMA (14), we identified an enrichment of genes at associated loci involved in pathways related to lipid metabolism and genital and digestive tract development at *P* < 0.05 after adjustment for multiple testing (Supplementary Material, Table S7). We did not observe evidence of tissue enrichment for genes in the associated loci (Supplementary Material, Fig. S4).

### We observed three signals at the previously reported *CUBN* locus

**Figure 1:**
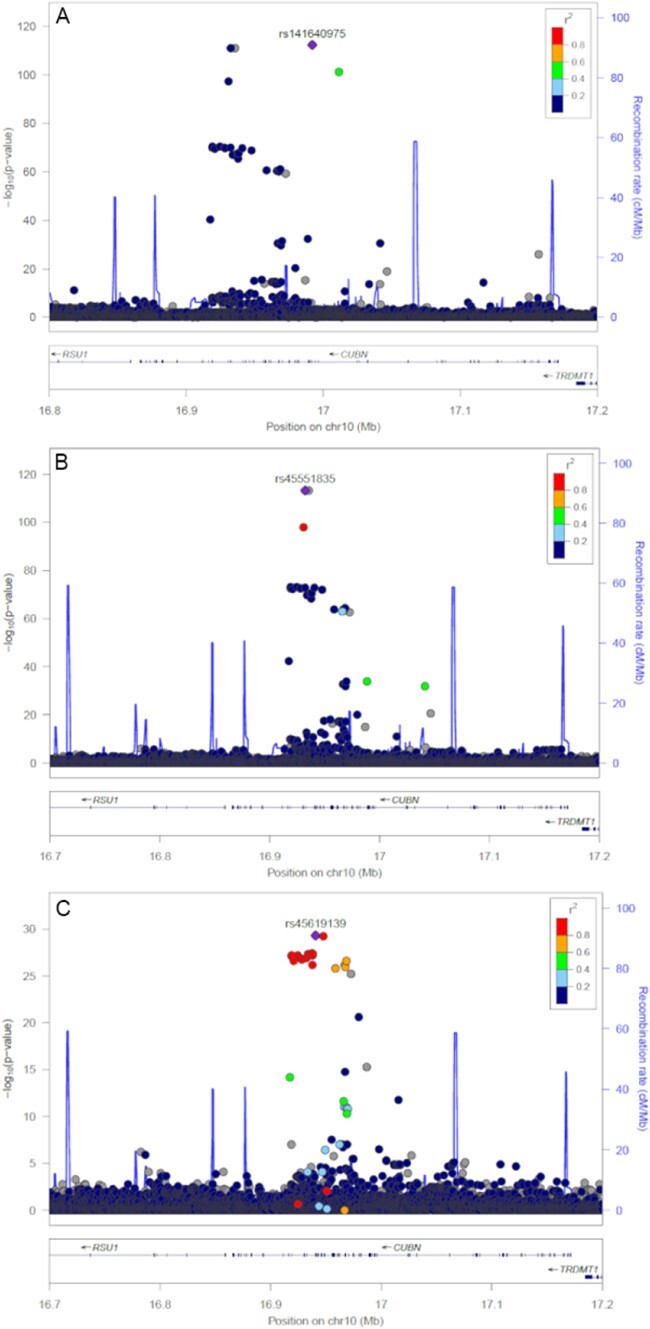
Significance of SNP associations for three independent signals at the CUBN locus. (**A**) Association of SNPs from the initial GWAS analysis. (**B**) Strength of SNP associations after the first round of conditional analysis. (**C**) Strength of SNP associations after the second round of conditional analysis.

We identified three independent SNPs associated with ACR at the *CUBN* locus (rs45619139, rs45551835 and rs141640975). These variants had common (rs45619139; MAF 10.1%), low (rs45551835; MAF 1.4%) and rare (rs141640975; MAF 0.3%) allele frequencies and were weakly correlated (low linkage disequilibrium). The minor alleles at the common, low-frequency and rare variants were associated with 0.06, 0.19 and 0.46 standard deviation higher ACR, respectively (Table 3 and Fig. 1). The common variant previously reported was rs1801239, but this association was abolished after the adjustment for stronger lead SNP rs45619139 in the UK Biobank (*r*^2^ = 0.78). The low-frequency (rs45551835) and rare (rs141640975) variants both alter the amino acid sequence of *CUBN* (g.16932384G>A, p.Ala2914Val) and (g.16992011G>A, p.Ala1690Val) respectively, and have previously been associated with variation in ACR.

**Table 3:** Independent signals identified at the CUBN locus on chromosome 10. Associations in the CUBN locus with ACR in UK Biobank whole cohort. Univariable results: each SNP tested independently. Multivariable results: all three SNPs tested in the same regression model

	**SNP**	**Position (b37)**	**Effect/other allele**	**Frequency effect allele**	**Model**	**Beta**	**SE**	***P***
	rs141640975	10:16992011	A/G	0.003	Univariable	0.463	0.022	4E-99
					Multivariable	0.470	0.022	3E-102
CUBN	rs45551835	10:16932384	A/G	0.014	Univariable	0.188	0.009	2E-90
					Multivariable	0.156	0.010	1E-56
	rs45619139	10:16940846	G/C	0.101	Univariable	0.059	0.004	1E-57
					Multivariable	0.040	0.004	7E-25

SNP = single-nucleotide polymorphism; b37 = build 37; SE = standard error; *P* = *P* value

### Haplotype analysis suggests alleles at the *CUBN* locus have additive effects on ACR

To test the effects of carrying more than one of the three *CUBN* variants, and whether in *cis* (one copy of the *CUBN* gene carrying two or three minor alleles) or *trans* (both copies of the *CUBN* gene carrying at least one minor allele), we estimated the haplotypes formed by the three SNPs. The correlation between the three SNPs was low. However, the *D*′ measure of linkage disequilibrium was high, suggesting that a limited number of recombination events have occurred between the three variants: *D*′ = 1 between rs141640975 and rs45551835 and between rs141640975 and rs45619139, and *D*′ = 0.92 between rs45551835 and rs45619139. The variants formed five out of a maximum of eight haplotypes (Table 4). As expected from its very low frequency, the minor allele (A) at the rare variant rs141640975 occurred on only one haplotype—together with the common alleles at the two other variants (G–C–A; frequency = 0.3%) and had the largest effect (0.48 SD). All four potential haplotypes formed by the common (rs45619139) and low-frequency (rs45551835) *CUBN* variants were detected—indicating that a recombination event must have occurred between them (or, less likely, a second identical mutation). However, the ACR-raising allele (A) at the low-frequency variant occurred much more frequently on the same haplotype with the ACR-raising allele at the common variant (G) (**A–G**–G; frequency = 1.3%), rather than with the ACR-lowering allele (C) (**A–C**–G; frequency = 0.1%; Table 4). The A–G–G haplotype was associated with 0.2 SDs higher ACR in contrast to the commonest haplotype, formed by the three common alleles (G–C–G). This effect was larger than that of the two haplotypes carrying only one of the ACR-raising alleles for the low-frequency or common variant (**A**–C–G and G–**G**–G), consistent with an additive effect of the two alleles, suggesting that the presence of the two alleles in *cis* on the same haplotype did not change their effects (Table 4).

**Table 4:** Haplotype associations with ACR based on the three SNPs in the CUBN locus. Effect sizes are given in standard deviations of inverse-normalized ACR and are relative to the baseline haplotype group formed by the three common alleles of the three SNPs. The *χ*^2^ test statistics and *P* values for each haplotype correspond to the significance of the association when compared against all other haplotypes pooled. Alleles are ordered across haplotypes based on genomic position and represent (1) the low-frequency variant rs45551835, (2) the common variant rs45619139 and (3) the rare variant rs141640975

**Haplotype**	**Frequency**	**Additive effect**	**95% CI**	*χ* ^2^	***P***
G–C–G (000)	0.895	*REF*	*REF*	352.7	1E-78
G–G–G (010)	0.086	0.039	0.031, 0.047	72.6	2E-17
A–G–G (110)	0.013	0.201	0.181, 0.221	370.1	2E-82
G–C–A (001)	0.003	0.482	0.437, 0.527	421.9	2E-94
A–C–G (100)	0.001	0.138	0.065, 0.211	15.2	1E-04

95% CI = 95% confidence interval; *χ*^2^ = chi-squared test statistic; *P* = *P* value

### Carriers of ACR-raising *CUBN* alleles have higher disease frequency

**Figure 2:**
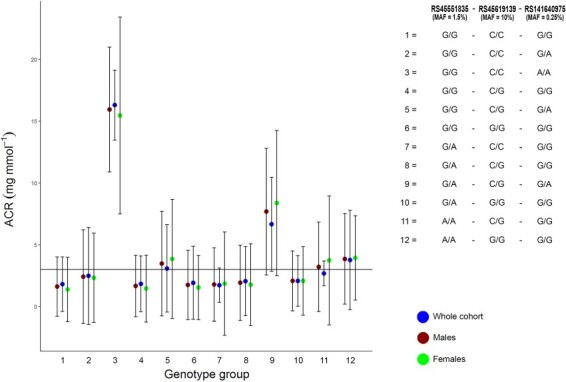
ACR by *CUBN* genotype group. ACR mean values and standard deviations by genotype group based on SNPs in the *CUBN* locus. Solid black line is the clinical threshold for microalbuminuria.

We next classified UK Biobank individuals into 12 groups based on genotype combinations of the three *CUBN* variants (Supplementary Material, Table S8 and Fig. S2). We tested the hypothesis that people with clinically classifiably microalbuminuria partly based on their *CUBN* genetics would have a lower frequency of vascular-related diseases in contrast to those without ACR-raising alleles at the *CUBN* locus. We noted that for some very rare *CUBN* genotype combinations, the majority of individuals would be classified as having microalbuminuria. For example, of the 25 individuals heterozygous at each of the three *CUBN* SNPs (<0.01% of the UK Biobank study), 21 (84%) had an ACR value that would classify them as having microalbuminuria (*P* < 0.001 Fisher’s exact test, in contrast to individuals carrying no ACR raising alleles at the *CUBN* locus) (Fig. 2).

We next selected all UK Biobank individuals above the clinical threshold for microalbuminuria to quantify the extent to which those with at least one ACR-raising allele at *CUBN* would have a lower frequency of vascular-related disease in contrast to those without a *CUBN-*raising allele. Of the 40 491/437027 individuals above the clinical threshold for microalbuminuria, 31 592 carried no ACR-raising alleles at the *CUBN* locus. Contrary to our hypothesis that *CUBN* ACR-raising alleles are benign for vascular-related diseases, people above the clinical threshold and carrying ACR-raising alleles at the *CUBN* locus had a higher frequency of CAD (carriers 11.2% vs non-carriers 10.2%, chi-squared *P =* 0.005), T2D (carriers 6.7% vs non-carriers 5.7%, chi-squared *P* < 0.001) and stroke (carriers 3.4% vs non-carriers 2.9%, chi-squared *P =* 0.006, Supplementary Material, Table S8).

### Additional ACR–diabetes interaction effects at the *CUBN* locus observed

We performed interaction analyses to test whether the 62 lead variants had stronger effects in individuals with diabetes as previously observed for the *CUBN* locus (5,8). We replicated the statistical interactions between the common and rare *CUBN* signals and diabetes status. In people with diabetes, each copy of the minor allele for the common variant rs45619139 was associated with a 0.12 SD (95% CI: 0.08–0.15) effect on ACR (inverse-normalized). The observed effect in people without diabetes was 0.06 SD (95% CI: 0.05–0.06) (*P*_interaction_ = 1 × 10^−5^) (Table 5 and Supplementary Material, Table S9). The minor alleles at the low-frequency and rare *CUBN* variants also showed evidence of statistical interaction with diabetes status, with larger effects on ACR in people with diabetes (rs45551835 *P*_interaction_ = 6 × 10^−7^; rs141640975 *P*_interaction_ = 4 × 10^−5^). These types of interaction are prone to statistical artifacts, especially when dichotomizing groups of people (15). We recently developed a negative control method based on a computational optimization algorithm to assess the likelihood of such artifacts (16) (url: https://github.com/drarwood/gags)—after 1000 repeated interaction analyses of groups of individuals sampled to have the same means and standard deviations of ACR as individuals with and without a diagnosis of diabetes, we observed an empirical *P* value ≤ 0.02 (19 of 1000 tests were more significant than the observed interaction at most). This suggests that the interaction was unlikely to be a statistical artifact of the differences in the two underlying distributions of ACR (Table 5 and Supplementary Material, Fig. S5). After accounting for multiple testing, we found no evidence of interaction for the remaining 59 index variants outside of the *CUBN* locus with main effects on ACR (Supplementary Material, Table S10).

**Table 5:** Associations of the three statistically independent SNPs at the CUBN locus within individuals with diabetes and without diabetes. The interaction term with diabetes is presented. Empirical *P* refers to the control experiment that involved the repeated sampling of two groups (×1000) matched on the distributions of ACR observed in individuals with and without diabetes prior to interaction analysis of the dummy group variable

		**Individuals with diabetes**			**Individuals without diabetes**			**Interaction term**			
**SNP**	**Effect allele**	**Beta**	**SE**	***P***	**Beta**	**SE**	***P***	**Beta**	**SE**	***P***	**Empirical *P***
rs45551835	A	0.115	0.044	3.8E-16	0.179	0.010	1.4E-78	0.213	0.043	6E-07	0.001
rs45619139	G	0.201	0.017	4.7E-11	0.055	0.004	3.7E-49	0.075	0.017	1E-05	0.002
rs141640975	A	0.781	0.107	2.6E-13	0.449	0.022	1.3E-89	0.426	0.104	4E-05	0.020

SNP = single-nucleotide polymorphism; SE = standard error; *P* = *P* value

When combining variants into a weighted GRS (excluding *CUBN* variants), we observed evidence of interaction with diabetes status, with the GRS having larger effects in people with diabetes (*P*_interaction_ = 2 × 10^−6^). After random sampling (described previously), we observed an empirical *P*_interaction_ = 0.11, suggesting that the interaction may not be specific to diabetes (Supplementary Material, Fig. S5). This T2D interaction may be a feature of metabolic disease in general because we also identified evidence of interaction between the ACR GRS and other diseases—all with stronger effects in cases than controls: hypertension (GRS *β*_cases_ = 0.036, 95% CI: 0.034–0.038; GRS *β*_controls_ = 0.025, 95% CI: 0.023–0.027; *P*_interaction_ = 2 × 10^−18^), CKD (GRS *β*_cases_ = 0.043, 95% CI: 0.030–0.056; GRS *β*_controls_ = 0.031, 95% CI: 0.030–0.033; *P*_interaction_ = 4 × 10^−05^), CAD (GRS *β*_cases_ = 0.034, 95% CI: 0.030–0.039; GRS *β*_controls_ = 0.032, 95% CI: 0.031–0.034; *P*_interaction_ = 3 × 10^−02^) and stroke (GRS *β*_cases_ = 0.039, 95% CI: 0.030–0.048; GRS *β*_controls_ = 0.032, 95% CI: 0.031–0.034; *P*_interaction_ = 2 × 10^−02^). In contrast to a previous study (5), we found no evidence of a gene–diabetes interaction at the *HS6ST1* locus (*P*_interaction_ *=* 0.76) or *RAB38/CTSC* locus (*P*_interaction_ = 0.06).

## Discussion

We performed a GWAS of ACR in 437 027 individuals from the UK Biobank and identified 62 SNPs in 56 loci associated with increased ACR. Of these, we identified 20 associations not previously known, of which 9 reached a stricter significance threshold of *P* < 6 × 10^−9^. Prior to the availability of the UK Biobank data, only one of these 56 loci (*CUBN*) was known to be associated with variation in ACR in individuals of European ancestry (5,6,8). Recent analysis, of smaller sets of unrelated individuals from the UK Biobank, identified 46 and 22 variants associated with ACR. The 62 variants we identified include 41/46 loci reported by the recent Haas *et al.* analysis (11). The five detected by Haas *et al.* but not us, despite our larger sample size, fell below the GWAS threshold of *P* < 5 × 10^−8^ (*P* values ranged between 1.5 × 10^−5^ and 4.9 × 10^−8^). This difference may reflect sampling error between our analyses. A more recent analyses of the UK Biobank data by Zanetti *et al.* (12) identified 19 associations with urinary ACR, but this analysis identified 22 variants after splitting the data into a discovery and replication dataset. These three analyses of the same data show the value in different groups analyzing large genetic datasets with different approaches.

A higher GRS for ACR was associated with higher risk of being above the clinical threshold for ACR. Like the Haas *et al.* study, we showed that people with a high ACR GRS were at higher risk of hypertension in contrast to those with a lower ACR, but there was no association with other diseases, including diabetes.

Our analysis of individual variants suggested that a higher genetic ACR results from a wide range of pathogenic mechanisms. Twenty of 62 ACR-associated SNPs have been previously associated at genome-wide significance with metabolic, inflammatory and vascular diseases and related traits. We identified over 200 separate ACR-SNP–second trait association entries in the NHGRI GWAS catalog (17), although over half included highly pleiotropic *GCKR* variants in LD with the our signal in *SNX17* (18). With the exception of the *SNX17* signal near *GCKR*, there were notably few that were known blood pressure, diabetes or CAD signals, and of these, they were not the most strongly associated variants for these traits suggesting that the variants identified as associated with ACR are likely pleiotropic. The only T2D variant apart from that near *GCKR* associated with ACR is that in *ARL15*, where the ACR-raising allele is associated with higher risk of T2D, but with a much lower odds ratio (1.06 [95% CI: 1.04–1.09)] (19) than many other known T2D variants. The ACR-raising allele at *ARL15* is also associated with apparently paradoxical effects on body composition (20) and poorer kidney function as measured by eGFR (21). That 20 of the 62 ACR-associated signals were previously known signals for a wide variety of traits, together with our pathway analyses implicating lipid metabolism and gut and genital development suggests a wide variety of mechanisms involved in normal variation in ACR. In addition, we note that 19/20 variant–ACR associations we present as novel are available in a recent meta-analysis of eGFR by Wuttke *et al.* (21). Of these, 16/19 effect estimates are directionally consistent with eGFR, with 10/16 at *P* < 0.05 and 4/16 at *P* < 5 × 10^−8^.

In contrast to previous studies, we performed a more extensive analysis of the previously reported *CUBN* locus (4,5). We showed that the low-frequency ACR-raising allele at rs45551835 most often occurs in *cis* with the more common ACR-raising allele at rs45619139, but this haplotype was associated with ACR consistent with the additive effects of the two alleles.

The association with hypertension indicates that disease processes underlie a high ACR GRS, rather than benign effects on kidney reabsorption and means that it would be inappropriate to tailor the clinical threshold to a person’s GRS for ACR. This is likely to be the case even for people carrying ACR-raising genotypes at the *CUBN* locus. These alleles might have been expected to be benign on disease risk, but for individuals above the microalbuminuria threshold our results provide evidence of higher frequencies of T2D, coronary heart disease and stroke among *CUBN* allele carriers in contrast to non-carriers*.*

The availability of individual level data from a large study also allowed us to test for gene–diabetes status interactions more extensively than before. Teumer *et al.* (2016) and Ahluwalia *et al.* (2019) showed that the ACR-raising minor-allele in *CUBN* had a 3- to 4-fold effect in people with diabetes in contrast to controls. Teumer *et al.* also reported larger effects in individuals with diabetes for variants in the *HS6ST1* and *RAB38/CTSC* loci. We tested these reported interactions and performed negative control experiments to control for the different distributions of ACR between people with and without diabetes (15). We replicated the previously reported interactions for the common and rare signal at the *CUBN* locus but did not find evidence of gene–diabetes interactions or main effects at the *HS6ST1* or *RAB38/CTSC* loci.

Our analysis had a number of strengths, including the availability of individual level data from a single large-study that provided homogeneous measures of ACR. Access to individual level data facilitated analyses that would otherwise be difficult to perform, including haplotype analysis, disease prevalence among rare genotype groups and interaction analysis with follow-up negative control experiments.

The main limitations of our study include the cross-sectional nature of the associations with disease prevalence. We are presently limited in our ability to evaluate the impact of ACR on disease outcomes prospectively in the UK Biobank within individuals who do not report having kidney disease. Second, the rare and low-frequency nature of the genetic variants with the largest effects mean replication of these signals will be difficult in many studies given relatively small sample sizes. Third, we calculated ACR in individuals with albumin levels below assay detection limits. Albumin levels were set to study specific limits of detection—an approach previously used by other GWAS analyses of ACR (5). Finally, further work needs to be undertaken in other ethnicities to determine whether the findings in our study replicate in other ancestries.

In conclusion, we have performed one of the largest genetic association studies on ACR and have gained further insight into the biological causes and clinical implications of raised ACR.

## Materials and Methods

### Study individuals

We used 437 027 UK Biobank individuals that had measures of ACR and were classified as of European ancestry through principal components analyses and a *k*-means clustering approach using the first four principal components and 1000 Genomes Project samples as reference ancestral centers.

### Albumin–creatinine ratio in UK Biobank

Measures of ACR were derived using urinary levels of albumin and creatinine. Albumin was measured in the UK Biobank samples using the immuno-turbidimetric analysis method (Randox Biosciences, UK) while creatinine was measured using the enzymatic analysis method (Beckman Coulter, UK). If albumin was <6.7 mg/L (the assay detection level in UK Biobank) then albumin was set at 6.7 mg/L prior to the calculation of the ratio, an approach consistent with previous studies (5).

### Albumin–creatinine ratio in EXTEND

Albumin and creatinine were measured in samples using immuno-turbidimetric and enzymatic methods, respectively. If albumin was < 2.9 mg/L (the assay detection level), then albumin was set at 2.9 mg/L prior to the calculation of the ratio.

### Genome-wide association analysis

Genetic associations for inverse-normalized ACR were tested using a linear-mixed model approach, implemented in BOLT-LMM (22). Models were adjusted for baseline age, sex, study center and genotyping array. Imputed genotypes from the Haplotype Reference Consortium (HRC) from the UK Biobank were used (23). Variants with imputation quality (INFO) < 0.3 or MAF < 0.1% were excluded. After quality control, 12 082 474 variants for association analysis remained. Lead SNPs were defined as those with the smallest *P* value. Locus boundaries were defined using a ±0.5 Mb distance from the lead SNP. Conditional analysis was performed by subsequently adding all lead SNPs for each locus as covariates.

### Lookups of associations using summary statistics from the CKDGen consortium

We downloaded summary statistics from the latest meta-analysis of ACR performed by CKDGen consortium to enable a comparison of directions of estimated effects for SNPs associated with ACR in the UK Biobank (5). We used proxies with *r*^2^ > 0.8 within 500 kb if unavailable in CKDGen.

### Variation explained and validation of GRS in the EXTEND study

We used the Exeter 10 000 (EXTEND) study to calculate the variance explained by discovered genetic associations in an independent cohort. EXTEND is a population-based study in the South West of England. Genotyping was performed using the Illumina Infinium Global Screening Array. Imputation of genotypes was performed using the Haplotype Reference Consortium (HRC) imputation reference panel (24). Analyses were based on 5679 individuals with genotype data and measures of ACR. Association analyses were carried out using RVTEST, adjusting for relatedness and ancestry through a genomic relationship matrix (25).

### GRS derivation for raised ACR

A weighted ACR GRS was calculated for each participant using the index variants identified from the UK Biobank analysis. Dosages were re-coded to ACR-increasing alleles prior to weighting using the respective effect sizes observed in the UK Biobank. A weighted score was subsequently calculated as the sum of the weighted dosages (Eq. 1) prior to re-scaling to reflect the number of ACR increasing alleles (Eq. 2).

(1) \begin{equation*} \mathrm{Weighted}\ \mathrm{score}=\beta1\ \times\ SNP1\ +\ \beta2 \ \times\ SNP2\ + \ \ldots\ \beta \mathrm{n}\ \times\ \mathrm{SNPn}. \end{equation*}

(2) \begin{equation*} \mathrm{GRS}=\frac{\mathrm{Weighted}\ \mathrm{score}\times \mathrm{n}\ \mathrm{SNPs}}{\sum \beta} \end{equation*}

### Testing the ACR GRS against risk of clinically defined microalbuminuria

We tested whether a higher ACR GRS was associated with being above the 3-mg/mmol clinical threshold (NICE, https://www.nice.org.uk/guidance/cg182) for microalbuminuria using logistic regression.

### Testing whether a high ACR GRS is associated with diabetes and vascular-related disease

We used logistic regression to test the combined role of ACR-associated variants in five diseases related to vascular dysfunction and diabetes in the UK Biobank—hypertension, T2D, coronary heart disease (CAD), CKD and stroke. Disease definitions were derived using a combination of questionnaire data, hospital episode statistics and interviews. Hypertension was defined as a systolic blood pressure of >140 mmHg, or a diastolic blood pressure of > 90 mmHg or the report of blood pressure medication usage using the baseline UK Biobank questionnaire. T2D was defined through self-report of diabetes using the UK Biobank questionnaire at baseline, > 35 years of age and without reporting of insulin use within the first year of diagnosis. We excluded individuals reporting diabetes diagnosed less than 1 year prior to baseline data collection (*N* = 1757) to exclude those who may be on insulin treatment within the first year of diagnosis and therefore could have other forms of diabetes. Incident cases (relative to UK Biobank baseline visit) of T2D were included using Hospital Episode Statistics (HES) data (from ICD10 code: E11.^*^). In addition, having any form of diabetes was defined for individuals who reported being informed of having the disease by their doctor (UK Biobank data field 2443). CAD was defined from HES and self-reported data from the UK Biobank questionnaire at baseline. Reporting of angina and/or myocardial infarction was used as criteria. CKD was defined using relevant primary and secondary ICD9 (580–629) and ICD10 codes (N00 to N99) available from HES data. Stroke was defined using codes 1583 (ischemic stroke), 1081 (stroke), 1086 (subarachnoid hemorrhage) and 1491 (brain hemorrhage) from clinic nurses’ codes for non-cancer illness (UK Biobank data field 20002).

### Investigating the overlap of loci associated with ACR with other traits from previous genome-wide association studies

We downloaded association statistics from the NHGRI-EBI GWAS Catalog (17). We looked up lead SNPs for ACR and proxies (*r*^2^ > 0.8) against SNPs catalogued with *P* value <5 × 10^−8^.

### Colocalization analysis of ACR-associated SNPs associated with other traits

We performed colocalization analysis to determine the likelihood of shared causal variants at associated loci that overlap for other traits in the GWAS catalog. We used summary statistics for SNPs 500 Kb on either side of the lead ACR-associated variant using publicly available GWAS data (16,26–32), aligning the effects to the ACR effect alleles. We used the coloc.abf function to estimate the probability of each locus sharing a causal variant.

### Gene-set and tissue enrichment analyses

Gene-set analyses and tissue expression analyses were performed using MAGMA (14) as implemented in the online Functional Mapping and Annotation of Genome-Wide Association Studies (FUMA) tool (33).

### Haplotype estimation and testing of associated SNPs in the CUBN locus

Estimation and testing of haplotypes were performed using UNPHASED (version 3.1.7) (34). Genotypes were converted to best-guess genotypes (0, 1, or 2) prior to haplotype estimation. Effect estimates were made relative to the reference haplotype comprising the common alleles. This analysis was performed in a subset of 367 882 unrelated UK Biobank individuals (<3rd degree relatives).

#### Interaction analyses

We performed interaction analyses for both novel and previously reported (5) SNPs to test for differences in effect sizes between diabetes cases and controls. These analyses were performed in the unrelated subset of 367 882 UK Biobank individuals. Of these, we classified 17 671 as having some form of diabetes. We compared effect sizes in diabetes cases versus controls by fitting the multiplicative interaction model and testing if not equal to zero—specifically (Eq. 3)

(3) \begin{equation*}{\rm ACR}={\rm SNP}_{\rm ACR} + {\rm diabetes}_{case/control}+{\rm SNP}_{\rm ACR}\times {\rm diabetes}_{case/control} [+ {\rm covariates}] \end{equation*}

#### Negative control experiment to test specificity of interactions

We tested whether putative interactions were specific to the interacting condition (e.g. diabetes), or an artifact of the highly skewed distribution. Using a computational optimization (genetic) algorithm (url: https://github.com/drarwood/gags), we repeatedly sampled individuals from the UK Biobank to derive groups matched to the ACR distributions of diabetes cases and controls but randomized to diabetes status. We repeated this random sampling 1000 times and compared the results to the observed interaction (15).

## Supplementary Material

20190927_R2_Supplementary_material_clean_ddz243

## Data Availability

Summary results for the GWAS analyses performed will be available from http://www.t2diabetesgenes.org/data/ on publication of this manuscript.
