# Supplementary material for: A genome-wide association study implicates multiple mechanisms influencing raised urinary albumin–creatinine ratio
Source: Hum Mol Genet. 2019 Oct 20;28(24):4197–207. doi: 10.1093/hmg/ddz243 (PMC7246045; doi:10.1093/hmg/ddz243)

**Supplementary material table of content**

Page 2. Supplementary table 1. GWAS summary statistics for the 62 SNPs in 56 loci associated with ACR at P<5x10^-8^ in the UK Biobank.

Page 4. Supplementary table 2. Summary statistics for the 62 SNPs associated with ACR in the UK Biobank and their proxies in the publicly available CKDGen consortium GWAS data.

Page 7. Supplementary table 3. Summary statistics for the 62 SNPs in EXTEND.

Page 9. Supplementary table 4. Association between the ACR Genetic Risk Score (GRS) and risk of five common disease/outcomes.

Page 10. Supplementary table 5. ACR associated SNPs and their proxies previously reported in GWAS catalogue with details of relevant studies.

Page 18. Supplementary table 6. Results from COLOC analysis for ACR associated variants previously known to be associated with other traits.

Page 19. Supplementary table 7. Results of gene-set enrichment derived using MAGMA as implemented in FUMA.

Page 20. Supplementary table 7. UK Biobank individuals classified based on their combination of CUBN genotypes.

Page 22. Supplementary table 8. . Effect of associations in the CUBN locus with ACR in people with and without diabetes in the UK Biobank.

Page 23. Supplementary table 9. Results of interaction term for 62 ACR associated SNPs with diabetes status.

Page 25. Supplementary Figure 1.

Page 26. Supplementary Figure 2.

Page 27. Supplementary Figure 3.

Page 28. Supplementary Figure 4.

Page 29. Supplementary Figure 5.

**SUPPLEMENTARY TABLES**

**Supplementary Table 1.** GWAS summary statistics for the 62 SNPs in 56 loci associated with ACR at P<5x10^-8^ in the UK Biobank. Association signals not reported by Teumer *et al.* (2016), Haas *et al.* (2018), Ahluwalia *et al.* (2019) or Zanetti *et al.* (2019) are emboldened. Loci not previous reported highlighted in blue. *Chromosome. †Base pair position (build hg19). ‡Effect allele / other allele. §Effect allele frequency. ||Imputation quality score. **Standard error.

| **Locus** | **Nearest Gene** | **rsID** | **Chr^*^** | **Position**^†^ | **EA/OA**^‡^ | **EA Freq**^§^ | **INFO**^\|\|^ | **Beta** | **SE**** | **P-value** |
| --- | --- | --- | --- | --- | --- | --- | --- | --- | --- | --- |
| 1 | *PHC2/ZSCAN20* | rs12032996 | 1 | 33920586 | G/A | 0.838 | 1.000 | 0.016 | 0.003 | 3.7E-09 |
| 2 | *FOXD2* | rs1337526 | 1 | 47965130 | G/A | 0.801 | 0.996 | 0.027 | 0.003 | 6.4E-27 |
| 2 | *FOXD2* | rs6676159 | 1 | 47945825 | T/C | 0.380 | 0.986 | 0.015 | 0.003 | 1.8E-08 |
| 3 | *AK5* | rs11162351 | 1 | 77944732 | C/G | 0.604 | 0.995 | 0.012 | 0.002 | 2.5E-08 |
| 4 | *DPM3/KRTCAP2* | rs34257409 | 1 | 155131394 | T/G | 0.404 | 0.999 | 0.014 | 0.002 | 5.0E-12 |
| **4** | ***MSTO1*** | **rs35202981** | **1** | **155578042** | **G/A** | **0.139** | **0.960** | **0.017** | **0.003** | **1.4E-08** |
| 5 | *PRRC2C* | rs12727104 | 1 | 171423167 | G/A | 0.905 | 0.994 | 0.019 | 0.003 | 1.8E-08 |
| **6** | ***EDEM3*** | **rs78444298** | **1** | **184672098** | **G/A** | **0.980** | **1.000** | **0.045** | **0.007** | **1.4E-09** |
| 7 | *SNX17* | rs4665972 | 2 | 27598097 | T/C | 0.394 | 0.987 | 0.016 | 0.002 | 4.8E-14 |
| 8 | *PARTICL* | rs12714144 | 2 | 85754578 | A/T | 0.872 | 0.996 | 0.020 | 0.003 | 1.6E-11 |
| **9** | ***GPD2*** | **rs111688960** | **2** | **157599687** | **A/G** | **0.013** | **0.987** | **0.051** | **0.009** | **1.2E-08** |
| 10 | *ICA1L* | rs10207567 | 2 | 203714973 | C/G | 0.814 | 0.999 | 0.018 | 0.003 | 1.7E-12 |
| 11 | *CPS1* | rs1047891 | 2 | 211540507 | C/A | 0.684 | 1.000 | 0.017 | 0.002 | 4.7E-15 |
| 12 | *NYAP2* | rs183131780 | 2 | 226684886 | T/C | 0.002 | 0.912 | 0.201 | 0.024 | 3.2E-17 |
| 13 | *COL4A4* | rs35483183 | 2 | 227876687 | A/G | 0.123 | 0.990 | 0.021 | 0.003 | 2.6E-11 |
| 14 | *SPHKAP* | rs35924503 | 2 | 229131286 | C/T | 0.001 | 0.882 | 0.295 | 0.031 | 9.2E-21 |
| 15 | *MYL3* | rs6768627 | 3 | 46895376 | T/C | 0.069 | 0.991 | 0.023 | 0.004 | 8.1E-09 |
| 16 | *PRKCI* | rs112607182 | 3 | 170027407 | T/C | 0.075 | 0.922 | 0.028 | 0.004 | 9.8E-13 |
| 17 | *NMU* | rs3805382 | 4 | 56471551 | A/G | 0.710 | 0.987 | 0.012 | 0.002 | 3.8E-08 |
| 18 | *SHROOM3* | rs10023335 | 4 | 77358987 | T/C | 0.591 | 0.999 | 0.012 | 0.002 | 1.9E-09 |
| 19 | *NR3C2* | rs6535594 | 4 | 149132756 | A/G | 0.496 | 0.991 | 0.014 | 0.002 | 3.0E-12 |
| **20** | ***FAT1*** | **rs62342738** | **4** | **187656129** | **C/G** | **0.181** | **0.987** | **0.015** | **0.003** | **5.5E-09** |
| 21 | *FGR1* | rs189107782 | 4 | 190729009 | T/C | 0.002 | 0.737 | 0.307 | 0.025 | 4.4E-35 |
| 21 | *FGR1* | rs4109437 | 4 | 190769223 | A/G | 0.038 | 1.000 | 0.041 | 0.005 | 4.2E-14 |
| 22 | *ARL15* | rs4865796 | 5 | 53272664 | A/G | 0.692 | 1.000 | 0.015 | 0.002 | 7.6E-12 |
| 22 | *ARL15* | rs31226 | 5 | 53327571 | T/C | 0.394 | 0.987 | 0.013 | 0.002 | 1.9E-08 |
| 23 | *CWC27* | rs7731168 | 5 | 64296471 | C/G | 0.233 | 0.996 | 0.015 | 0.002 | 8.0E-10 |
| **24** | ***C5orf56*** | **rs11242113** | **5** | **131777234** | **A/G** | **0.188** | **0.994** | **0.016** | **0.003** | **1.6E-09** |
| **25** | ***KCNK5*** | **rs1544935** | **6** | **39124448** | **G/T** | **0.216** | **0.987** | **0.017** | **0.002** | **2.2E-11** |
| **26** | ***VEGFA*** | **rs3734692** | **6** | **43817791** | **T/A** | **0.310** | **0.990** | **0.016** | **0.002** | **1.8E-13** |
| 27 | *AHR* | rs4410790 | 7 | 17284577 | C/T | 0.633 | 1.000 | 0.024 | 0.002 | 1.7E-30 |
| 28 | *HOTTIP* | rs2023844 | 7 | 27243238 | A/G | 0.926 | 0.999 | 0.027 | 0.004 | 5.2E-12 |
| 29 | *DPY19L2P3/WIPF3* | rs9638860 | 7 | 29794937 | T/C | 0.260 | 0.982 | 0.017 | 0.002 | 2.1E-13 |
| **30** | ***AUTS2*** | **rs35692677** | **7** | **69902654** | **G/A** | **0.813** | **0.983** | **0.015** | **0.003** | **1.1E-08** |
| **31** | ***ZBTB10*** | **rs11990607** | **8** | **81363534** | **A/G** | **0.835** | **0.995** | **0.015** | **0.003** | **2.5E-08** |
| 32 | *TRIB1* | rs28601761 | 8 | 126500031 | C/G | 0.580 | 0.974 | 0.016 | 0.002 | 2.6E-14 |
| 33 | *CUBN* | rs141640975 | 10 | 16992011 | A/G | 0.003 | 1.000 | 0.452 | 0.020 | 4.8E-113 |
| 33 | *CUBN* | rs45551835 | 10 | 16932384 | A/G | 0.014 | 1.000 | 0.193 | 0.008 | 6.5E-114 |
| 33 | *CUBN* | rs45619139 | 10 | 16940846 | G/C | 0.101 | 0.993 | 0.040 | 0.004 | 5.0E-30 |
| **34** | ***MLLT10*** | **rs6482189** | **10** | **21889138** | **G/A** | **0.683** | **0.998** | **0.013** | **0.002** | **1.6E-09** |
| 35 | *ADO* | rs10995311 | 10 | 64564934 | C/G | 0.554 | 0.990 | 0.013 | 0.002 | 7.9E-10 |
| 36 | *C10orf11* | rs7898462 | 10 | 77894968 | G/C | 0.220 | 0.983 | 0.018 | 0.002 | 1.7E-13 |
| **37** | ***CYP26A1*** | **rs2068888** | **10** | **94839642** | **G/A** | **0.550** | **1.000** | **0.012** | **0.002** | **1.0E-09** |
| **38** | ***SBF2*** | **rs11042685** | **11** | **10262551** | **C/T** | **0.493** | **0.999** | **0.011** | **0.002** | **2.1E-08** |
| **39** | ***NUMA1*** | **rs7115200** | **11** | **71752160** | **G/T** | **0.440** | **1.000** | **0.012** | **0.002** | **1.4E-09** |
| **40** | ***OAF*** | **rs12790943** | **11** | **120058623** | **T/C** | **0.421** | **0.997** | **0.011** | **0.002** | **3.0E-08** |
| 41 | *CCT2* | rs2601006 | 12 | 69979517 | C/T | 0.657 | 0.996 | 0.015 | 0.002 | 9.9E-13 |
| **42** | ***NAV3*** | **rs10860332** | **12** | **78748014** | **A/G** | **0.414** | **0.982** | **0.011** | **0.002** | **4.4E-08** |
| **43** | ***DLEU1/BCMS*** | **rs3116613** | **13** | **51143055** | **G/T** | **0.211** | **0.997** | **0.014** | **0.002** | **3.9E-08** |
| 44 | *ZFP36L1* | rs4899263 | 14 | 69285264 | G/A | 0.469 | 0.990 | 0.012 | 0.002 | 6.3E-09 |
| 45 | *TYRO3* | rs28844285 | 15 | 41879640 | T/C | 0.595 | 0.989 | 0.014 | 0.002 | 1.2E-11 |
| 46 | *SPATA5L1* | rs60476496 | 15 | 45713801 | C/T | 0.745 | 1.000 | 0.018 | 0.002 | 9.2E-16 |
| 47 | *USP3* | rs146311723 | 15 | 63804507 | C/T | 0.175 | 0.996 | 0.017 | 0.003 | 4.3E-10 |
| 48 | *CYP1A1/CYP1A2* | rs2472297 | 15 | 75027880 | T/C | 0.265 | 1.000 | 0.027 | 0.002 | 1.7E-32 |
| **49** | ***WDR81*** | **rs550628400** | **17** | **1639795** | **G/A** | **0.006** | **0.894** | **0.075** | **0.013** | **2.1E-08** |
| 50 | *FBXL20* | rs2338796 | 17 | 37555627 | A/G | 0.670 | 0.993 | 0.012 | 0.002 | 1.1E-08 |
| 51 | *BAHCC1* | rs35572189 | 17 | 79419025 | G/A | 0.637 | 0.994 | 0.012 | 0.002 | 9.2E-09 |
| 52 | *TCF4/LINC01415* | rs784257 | 18 | 53397199 | T/C | 0.187 | 0.988 | 0.015 | 0.003 | 2.7E-09 |
| **53** | ***CYP2A7*** | **rs79600176** | **19** | **41392490** | **T/C** | **0.978** | **1.000** | **0.038** | **0.007** | **3.6E-08** |
| **54** | ***CCDC97*** | **rs56254331** | **19** | **41826020** | **A/C** | **0.831** | **0.999** | **0.017** | **0.003** | **7.9E-10** |
| 55 | *FUT1* | rs838142 | 19 | 49252151 | A/G | 0.723 | 0.997 | 0.016 | 0.002 | 1.3E-12 |
| **56** | ***ZBTB46*** | **rs11697610** | **20** | **62379531** | **G/A** | **0.387** | **0.984** | **0.012** | **0.002** | **1.9E-08** |

**Supplementary Table 2**. Summary statistics for the 62 SNPs associated with ACR in the UK Biobank and their proxies in the publicly available CKDGen consortium GWAS data if not available. *Chromosome of proxy SNP. †Base pair position (build hg19). ‡Linkage disequilibrium estimate with lead ACR. §Effect allele / other allele. Betas in the UK Biobank are based on inverse-normal ACR. Betas from the CKDGen consortium are based on logged ACR.

|  |  |  |  |  |  |  | **UK Biobank** | | | **CKDGen Consortium** | | |
| --- | --- | --- | --- | --- | --- | --- | --- | --- | --- | --- | --- | --- |
| **Locus** | **rsID** | **CDKGEN**  **Proxy** | **Proxy**  **Chr^*^** | **Proxy**  **Position**^†^ | **Proxy**  **LD r2**^‡^ | **EA/OA**^§^ | **Beta** | **SE** | **P-value** | **Beta** | **SE** | **P-value** |
| *PHC2/ZSCAN20* | rs12032996 |  |  |  |  | G/A | 0.016 | 0.003 | 3.7E-09 | 0.010 | 0.008 | 2.2E-01 |
| *FOXD2* | rs1337526 |  |  |  |  | G/A | 0.027 | 0.003 | 6.4E-27 | 0.015 | 0.007 | 2.2E-01 |
| *FOXD2* | rs6676159 | rs1591658 | 1 | 47962802 | 0.912 | T/A | 0.015 | 0.003 | 1.8E-08 | 0.008 | 0.006 | 4.3E-02 |
| *AK5* | rs11162351 |  |  |  |  | C/G | 0.012 | 0.002 | 2.5E-08 | -0.006 | 0.006 | 3.5E-01 |
| *DPM3/KRTCAP2* | rs34257409 | rs4276913 | 1 | 155131673 | 0.998 | G/A | 0.014 | 0.002 | 5.0E-12 | 0.020 | 0.006 | 9.6E-04 |
| *PRRC2C* | rs12727104 | rs16864515 | 1 | 171435542 | 0.985 | C/A | 0.019 | 0.003 | 1.8E-08 | 0.007 | 0.010 | 4.6E-01 |
| *SNX17* | rs4665972 | rs1260326 | 2 | 27730940 | 0.898 | T/C | 0.016 | 0.002 | 4.8E-14 | 0.022 | 0.006 | 2.7E-04 |
| *PARTICL* | rs12714144 | rs10202828 | 2 | 85762675 | 0.963 | C/T | 0.020 | 0.003 | 1.6E-11 | 0.018 | 0.010 | 5.9E-02 |
| *ICA1L* | rs10207567 | rs1971819 | 2 | 203705787 | 0.996 | C/G | 0.018 | 0.003 | 1.7E-12 | 0.015 | 0.008 | 5.2E-02 |
| *CPS1* | rs1047891 | rs715 | 2 | 211543055 | 0.930 | T/C | 0.017 | 0.002 | 4.7E-15 | 0.012 | 0.009 | 1.7E-01 |
| *COL4A4* | rs35483183 | rs7604486 | 2 | 227856332 | 0.978 | G/C | 0.021 | 0.003 | 2.6E-11 | 0.009 | 0.010 | 3.7E-01 |
| *MYL3* | rs6768627 |  |  |  |  | T/C | 0.023 | 0.004 | 8.1E-09 | 0.038 | 0.013 | 2.6E-03 |
| *NMU* | rs3805382 |  |  |  |  | A/G | 0.012 | 0.002 | 3.8E-08 | 0.010 | 0.007 | 1.2E-01 |
| *SHROOM3* | rs10023335 |  |  |  |  | T/C | 0.012 | 0.002 | 1.9E-09 | 0.011 | 0.006 | 8.1E-02 |
| *NR3C2* | rs6535594 |  |  |  |  | A/G | 0.014 | 0.002 | 3.0E-12 | 0.018 | 0.007 | 5.7E-03 |
| *FAT1* | rs62342738 | rs11132407 | 4 | 187656456 | 0.995 | G/T | 0.015 | 0.003 | 5.5E-09 | -0.013 | 0.011 | 2.4E-01 |
| *ARL15* | rs4865796 |  |  |  |  | A/G | 0.015 | 0.002 | 7.6E-12 | 0.005 | 0.006 | 4.6E-01 |
| *ARL15* | rs31226 |  |  |  |  | T/C | 0.013 | 0.002 | 1.9E-08 | 0.002 | 0.006 | 7.0E-01 |
| *CWC27* | rs7731168 | rs2278352 | 5 | 64267431 | 0.951 | G/A | 0.015 | 0.002 | 8.0E-10 | 0.001 | 0.007 | 8.8E-01 |
| *C5orf56* | rs11242113 | rs1012793 | 5 | 131781345 | 0.998 | C/G | 0.016 | 0.003 | 1.6E-09 | 0.022 | 0.008 | 3.9E-03 |
| *KCNK5* | rs1544935 |  |  |  |  | G/T | 0.017 | 0.002 | 2.2E-11 | 0.017 | 0.007 | 1.8E-02 |
| *VEGFA* | rs3734692 | rs9369427 | 6 | 43811430 | 0.878 | C/A | 0.016 | 0.002 | 1.8E-13 | 0.018 | 0.007 | 5.8E-03 |
| *AHR* | rs4410790 |  |  |  |  | C/T | 0.024 | 0.002 | 1.7E-30 | 0.001 | 0.007 | 8.6E-01 |
| *HOTTIP* | rs2023844 |  |  |  |  | A/G | 0.027 | 0.004 | 5.2E-12 | 0.036 | 0.014 | 8.7E-03 |
| *DPY19L2P3/WIPF3* | rs9638860 | rs17158386 | 7 | 29805361 | 0.955 | A/G | 0.017 | 0.002 | 2.1E-13 | 0.020 | 0.009 | 2.9E-02 |
| *AUTS2* | rs35692677 | rs12698905 | 7 | 69917440 | 0.954 | G/C | 0.015 | 0.003 | 1.1E-08 | 0.015 | 0.009 | 9.0E-02 |
| *ZBTB10* | rs11990607 |  |  |  |  | A/G | 0.015 | 0.003 | 2.5E-08 | 0.020 | 0.009 | 2.3E-02 |
| *MLLT10* | rs6482189 |  |  |  |  | G/A | 0.013 | 0.002 | 1.6E-09 | 0.004 | 0.009 | 7.0E-01 |
| *ADO* | rs10995311 | rs11592442 | 10 | 64533904 | 0.918 | T/A | 0.013 | 0.002 | 7.9E-10 | -0.002 | 0.006 | 7.8E-01 |
| *C10orf11* | rs7898462 | rs7915302 | 10 | 77895119 | 0.998 | C/T | 0.018 | 0.002 | 1.7E-13 | 0.004 | 0.007 | 5.5E-01 |
| *CYP26A1* | rs2068888 |  |  |  |  | G/A | 0.012 | 0.002 | 1.0E-09 | 0.0180 | 0.007 | 7.7E-03 |
| *SBF2* | rs11042685 | rs2197170 | 11 | 10247799 | 0.997 | G/A | 0.011 | 0.002 | 2.1E-08 | -0.001 | 0.006 | 8.4E-01 |
| *NUMA1* | rs7115200 | rs606799 | 11 | 71608582 | 0.876 | G/C | 0.012 | 0.002 | 1.4E-09 | 0.013 | 0.007 | 7.5E-02 |
| *OAF* | rs12790943 | rs10892547 | 11 | 120060793 | 0.904 | T/G | 0.011 | 0.002 | 3.0E-08 | 0.011 | 0.006 | 6.7E-02 |
| *CCT2* | rs2601006 |  |  |  |  | C/T | 0.015 | 0.002 | 9.9E-13 | -0.001 | 0.007 | 8.5E-01 |
| *NAV3* | rs10860332 |  |  |  |  | A/G | 0.011 | 0.002 | 4.4E-08 | -0.004 | 0.007 | 5.3E-01 |
| *DLEU1/BCMS* | rs3116613 | rs2408045 | 13 | 51142065 | 0.999 | T/C | 0.014 | 0.002 | 3.9E-08 | -0.006 | 0.007 | 4.2E-01 |
| *ZFP36L1* | rs4899263 |  |  |  |  | G/A | 0.012 | 0.002 | 6.3E-09 | 0.006 | 0.006 | 3.2E-01 |
| *TYRO3* | rs28844285 | rs4924556 | 15 | 41878208 | 0.998 | C/A | 0.014 | 0.002 | 1.2E-11 | 0.001 | 0.007 | 8.7E-01 |
| *SPATA5L1* | rs60476496 | rs12440038 | 15 | 45706739 | 0.998 | A/G | 0.018 | 0.002 | 9.2E-16 | 0.009 | 0.007 | 1.6E-01 |
| *USP3* | rs146311723 | rs17184782 | 15 | 63815479 | 0.902 | C/T | 0.017 | 0.003 | 4.3E-10 | -0.0001 | 0.008 | 9.9E-01 |
| *CYP1A1/CYP1A2* | rs2472297 |  |  |  |  | T/C | 0.027 | 0.002 | 1.7E-32 | 0.002 | 0.009 | 8.3E-01 |
| *BAHCC1* | rs35572189 | rs1478610 | 17 | 79422252 | 0.892 | G/A | 0.012 | 0.002 | 9.2E-09 | 0.016 | 0.009 | 5.4E-02 |
| *TCF4/LINC01415* | rs784257 |  |  |  |  | T/C | 0.015 | 0.003 | 2.7E-09 | 0.015 | 0.009 | 9.8E-02 |
| *CCDC97* | rs56254331 | rs15052 | 19 | 41813375 | 0.935 | T/C | 0.017 | 0.003 | 7.9E-10 | 0.009 | 0.013 | 4.8E-01 |
| *FUT1* | rs838142 | rs4021 | 19 | 49253261 | 0.994 | A/G | 0.016 | 0.002 | 1.3E-12 | 0.025 | 0.009 | 4.4E-03 |
| *ZBTB46* | rs11697610 | rs7273624 | 20 | 62385479 | 0.896 | A/G | 0.012 | 0.002 | 1.9E-08 | 0.002 | 0.006 | 7.6E-01 |

**Supplementary Table 3.** Summary statistics for the 62 SNPs in EXTEND. Betas are aligned to the ACR raising allele in UK Biobank. Beta estimates are based on inverse-normalised ACR.

|  |  | **EXTEND** | | | **UK BIOBANK** | | |
| --- | --- | --- | --- | --- | --- | --- | --- |
| **Locus** | **rsID** | **Beta** | **SE** | **P-value** | **Beta** | **SE** | **P-value** |
| *PHC2/ZSCAN20* | rs12032996 | -0.004 | 0.026 | 8.6E-01 | 0.016 | 0.003 | 3.7E-09 |
| *FOXD2* | rs1337526 | 0.019 | 0.023 | 4.0E-01 | 0.027 | 0.003 | 6.4E-27 |
| *FOXD2* | rs6676159 | -0.013 | 0.019 | 4.8E-01 | 0.015 | 0.003 | 1.8E-08 |
| *AK5* | rs11162351 | -0.002 | 0.019 | 9.1E-01 | 0.012 | 0.002 | 2.5E-08 |
| *DPM3/KRTCAP2* | rs34257409 | 0.013 | 0.019 | 4.7E-01 | 0.014 | 0.002 | 5.0E-12 |
| *MST01* | rs35202981 | 0.018 | 0.030 | 5.4E-01 | 0.017 | 0.003 | 1.4E-08 |
| *PRRC2C* | rs12727104 | 0.022 | 0.031 | 4.9E-01 | 0.019 | 0.003 | 1.8E-08 |
| *EDEM3* | rs78444298 | 0.084 | 0.069 | 2.2E-01 | 0.045 | 0.007 | 1.4E-09 |
| *SNX17* | rs4665972 | 0.001 | 0.018 | 9.5E-01 | 0.016 | 0.002 | 4.8E-14 |
| *PARTICL* | rs12714144 | -0.010 | 0.027 | 7.0E-01 | 0.020 | 0.003 | 1.6E-11 |
| *GPD2* | rs111688960 | 0.057 | 0.077 | 4.6E-01 | 0.051 | **0.009** | 1.2E-08 |
| *ICA1L* | rs10207567 | 0.032 | 0.023 | 1.7E-01 | 0.018 | 0.003 | 1.7E-12 |
| *CPS1* | rs1047891 | 0.033 | 0.019 | 8.9E-02 | 0.017 | 0.002 | 4.7E-15 |
| *NYAP2* | rs183131780 | 0.227 | 0.251 | 3.7E-01 | 0.201 | 0.024 | 3.2E-17 |
| *COL4A4* | rs35483183 | -0.003 | 0.029 | 9.3E-01 | 0.021 | 0.003 | 2.6E-11 |
| *SPHKAP* | rs35924503 | 0.673 | 0.409 | 1.0E-01 | 0.295 | 0.031 | 9.2E-21 |
| *MYL3* | rs6768627 | 0.038 | 0.035 | 2.7E-01 | 0.023 | 0.004 | 8.1E-09 |
| *PRKCI* | rs112607182 | 0.014 | 0.038 | 7.2E-01 | 0.028 | 0.004 | 9.8E-13 |
| *NMU* | rs3805382 | -0.038 | 0.021 | 6.8E-02 | 0.012 | 0.002 | 3.8E-08 |
| *SHROOM3* | rs10023335 | -0.012 | 0.018 | 5.3E-01 | 0.012 | 0.002 | 1.9E-09 |
| *NR3C2* | rs6535594 | -0.004 | 0.018 | 8.4E-01 | 0.014 | 0.002 | 3.0E-12 |
| *FAT1* | rs62342738 | -0.028 | 0.024 | 2.3E-01 | 0.015 | 0.003 | 5.5E-09 |
| *FGR1* | rs189107782 | 0.221 | 0.339 | 5.1E-01 | 0.307 | 0.025 | 4.4E-35 |
| *FGR1* | rs4109437 | -0.017 | 0.049 | 7.3E-01 | 0.041 | 0.005 | 4.2E-14 |
| *ARL15* | rs4865796 | 0.027 | 0.020 | 1.7E-01 | 0.015 | 0.002 | 7.6E-12 |
| *ARL15* | rs31226 | -0.031 | 0.019 | 9.4E-02 | 0.013 | 0.002 | 1.9E-08 |
| *CWC27* | rs7731168 | -0.002 | 0.022 | 9.1E-01 | 0.015 | 0.002 | 8.0E-10 |
| *C5orf56* | rs11242113 | -0.006 | 0.023 | 8.1E-01 | 0.016 | 0.003 | 1.6E-09 |
| *KCNK5* | rs1544935 | 0.043 | 0.022 | 5.6E-02 | 0.017 | 0.002 | 2.2E-11 |
| *VEGFA* | rs3734692 | 0.038 | 0.020 | 5.4E-02 | 0.016 | 0.002 | 1.8E-13 |
| *AHR* | rs4410790 | -0.010 | 0.019 | 5.9E-01 | 0.024 | 0.002 | 1.7E-30 |
| *HOTTIP* | rs2023844 | 0.076 | 0.035 | 3.1E-02 | 0.027 | 0.004 | 5.2E-12 |
| *DPY19L2P3/WIPF3* | rs9638860 | -0.013 | 0.021 | 5.3E-01 | 0.017 | 0.002 | 2.1E-13 |
| *AUTS2* | rs35692677 | 0.003 | 0.024 | 9.1E-01 | 0.015 | 0.003 | 1.1E-08 |
| *ZBTB10* | rs11990607 | 0.057 | 0.024 | 1.7E-02 | 0.015 | 0.003 | 2.5E-08 |
| *TRIB1* | rs28601761 | 0.012 | 0.019 | 5.3E-01 | 0.016 | 0.002 | 2.6E-14 |
| *CUBN* | rs141640975 | 0.459 | 0.189 | 1.5E-02 | 0.452 | 0.020 | 4.8E-113 |
| *CUBN* | rs45551835 | 0.208 | 0.079 | 8.8E-03 | 0.193 | 0.008 | 6.5E-114 |
| *CUBN* | rs45619139 | 0.131 | 0.030 | 1.2E-05 | 0.040 | 0.004 | 5.0E-30 |
| *MLLT10* | rs6482189 | 0.015 | 0.019 | 4.3E-01 | 0.013 | 0.002 | 1.6E-09 |
| *ADO* | rs10995311 | 0.008 | 0.019 | 6.5E-01 | 0.013 | 0.002 | 7.9E-10 |
| *C10orf11* | rs7898462 | 0.010 | 0.023 | 6.8E-01 | 0.018 | 0.002 | 1.7E-13 |
| *CYP26A1* | rs2068888 | 0.020 | 0.018 | 2.8E-01 | 0.012 | 0.002 | 1.0E-09 |
| *SBF2* | rs11042685 | -0.004 | 0.018 | 8.3E-01 | 0.011 | 0.002 | 2.1E-08 |
| *NUMA1* | rs7115200 | -0.017 | 0.020 | 4.1E-01 | 0.012 | 0.002 | 1.4E-09 |
| *OAF* | rs12790943 | 0.005 | 0.018 | 8.0E-01 | 0.011 | 0.002 | 3.0E-08 |
| *CCT2* | rs2601006 | 0.023 | 0.019 | 2.3E-01 | 0.015 | 0.002 | 9.9E-13 |
| *NAV3* | rs10860332 | -0.004 | 0.019 | 8.2E-01 | 0.011 | 0.002 | 4.4E-08 |
| *DLEU1/BCMS* | rs3116613 | -0.005 | 0.022 | 8.2E-01 | 0.014 | 0.002 | 3.9E-08 |
| *ZFP36L1* | rs4899263 | 0.022 | 0.018 | 2.2E-01 | 0.012 | 0.002 | 6.3E-09 |
| *TYRO3* | rs28844285 | 0.043 | 0.019 | 2.2E-02 | 0.014 | 0.002 | 1.2E-11 |
| *SPATA5L1* | rs60476496 | 0.003 | 0.021 | 8.8E-01 | 0.018 | 0.002 | 9.2E-16 |
| *USP3* | rs146311723 | -0.005 | 0.025 | 8.5E-01 | 0.017 | 0.003 | 4.3E-10 |
| *CYP1A1/CYP1A2* | rs2472297 | 0.034 | 0.022 | 1.3E-01 | 0.027 | 0.002 | 1.7E-32 |
| *WDR81* | rs550628400 | -0.139 | 0.143 | 3.3E-01 | 0.075 | 0.013 | 2.1E-08 |
| *FBXL20* | rs2338796 | 0.020 | 0.022 | 3.7E-01 | 0.012 | 0.002 | 1.1E-08 |
| *BAHCC1* | rs35572189 | 0.013 | 0.021 | 5.2E-01 | 0.012 | 0.002 | 9.2E-09 |
| *TCF4/LINC01415* | rs784257 | 0.009 | 0.023 | 6.9E-01 | 0.015 | 0.003 | 2.7E-09 |
| *CCDC97* | rs56254331 | 0.040 | 0.026 | 1.3E-01 | 0.017 | 0.003 | 7.9E-10 |
| *CYP2A7* | rs79600176 | 0.068 | 0.064 | 2.9E-01 | 0.038 | 0.007 | 3.6E-08 |
| *FUT1* | rs838142 | 0.042 | 0.021 | 4.4E-02 | 0.016 | 0.002 | 1.3E-12 |
| *ZBTB46* | rs11697610 | -0.025 | 0.019 | 1.8E-01 | 0.012 | 0.002 | 1.9E-08 |

**Supplementary Table 4.** Association between the ACR Genetic Risk Score (GRS) and risk of five common disease/outcomes (OR per standard deviation of the GRS). *Odds ratio. †95% confidence intervals. Results presented show the effects of the GRS in terms of per-unit increase and in terms of per-standard deviation increase.

|  | **GRS** | | | | |  |  | **GRS with *CUBN* SNPs excluded** | | | |
| --- | --- | --- | --- | --- | --- | --- | --- | --- | --- | --- | --- |
|  | **Per unit increase** | | **Per SD increase** | | | **Per unit increase** | | | **Per SD Increase** | | |
| **Disease / Outcome** | **OR^*^** | **95% CI**^†^ | **OR^*^** | **95% CI**^†^ | **P-value** | **OR^*^** | **95% CI**^†^ | | **OR^*^** | **95% CI**^†^ | **P-value** |
| Hypertension | 1.013 | 1.010 – 1.016 | 1.029 | 1.022 - 1.036 | 1.6E-16 | 1.013 | 1.010 – 1.016 | | 1.032 | 1.025 - 1.039 | 5.2E-19 |
| Stroke | 1.011 | 1.001 – 1.022 | 1.026 | 1.002 - 1.049 | 2.7E-02 | 1.008 | 0.998 – 1.017 | | 1.018 | 0.996 - 1.042 | 1.2E-01 |
| Type 2 Diabetes | 1.008 | 1.000 – 1.017 | 1.019 | 1.000 - 1.037 | 4.5E-02 | 1.007 | 1.000 – 1.015 | | 1.018 | 1.000 - 1.037 | 5.3E-02 |
| Coronary Artery Disease | 1.004 | 0.999 – 1.010 | 1.009 | 0.997 - 1.020 | 1.3E-01 | 1.001 | 0.996 – 1.006 | | 1.003 | 0.991 - 1.015 | 6.2E-01 |
| Chronic Kidney Disease | 0.996 | 0.982 – 1.010 | 0.991 | 0.961 - 1.023 | 5.9E-01 | 0.997 | 0.984 – 1.010 | | 0.993 | 0.962 - 1.024 | 6.4E-01 |

**Supplementary Table 5.** ACR associated SNPs and their proxies previously reported in GWAS catalogue with details of relevant studies. *Linkage disequilibrium estimate with lead ACR. †Effect allele. ‡Odds ratio or beta. *indicates lead SNP has not previously been reported to be associated with ACR. Accessed: 28/02/2018.

| **ACR**  **SNP** | **Nearest**  **Gene** | **GWAS**  **SNP** | **GWAS**  **R2^*^** | **GWAS**  **Author** | **GWAS**  **Year** | **GWAS**  **Trait** | **GWAS**  **EA**^†^ | **GWAS**  **OR / BETA**^‡^ | **GWAS**  **P-value** |
| --- | --- | --- | --- | --- | --- | --- | --- | --- | --- |
| rs34257409 | *DPM3/KRTCAP2* | rs10908458 | 0.982 | Chambers JC | 2011 | Liver enzyme levels (gamma-glutamyl transferase) | T | 3.700 | 2.0E-15 |
| rs4665972 | *SNX17* | rs780094 | 0.862 | Willer CJ | 2008 | Triglycerides | T | 8.590 | 6.0E-32 |
| rs4665972 |  | rs780094 | 0.862 | Ridker PM | 2008 | C-reactive protein | A | 0.140 | 7.0E-15 |
| rs4665972 |  | rs780094 | 0.862 | Kathiresan S | 2008 | Triglycerides | T | 0.130 | 3.0E-14 |
| rs4665972 |  | rs780094 | 0.862 | Aulchenko YS | 2008 | Triglycerides | G | 0.100 | 3.0E-20 |
| rs4665972 |  | rs780094 | 0.862 | Kolz M | 2009 | Uric acid levels | T | 0.050 | 1.0E-09 |
| rs4665972 |  | rs780094 | 0.862 | Suhre K | 2011 | Metabolic traits | T | 0.101 | 6.0E-53 |
| rs4665972 |  | rs780094 | 0.862 | Kristiansson K | 2012 | Metabolic syndrome | A | 0.130 | 6.0E-20 |
| rs4665972 |  | rs780094 | 0.862 | O'Seaghdha CM | 2013 | Calcium levels | T | 0.017 | 1.0E-10 |
| rs4665972 |  | rs780094 | 0.862 | Coram MA | 2013 | Triglycerides | C | 0.069 | 7.0E-09 |
| rs4665972 |  | rs780094 | 0.862 | Hwang JY | 2014 | Fasting plasma glucose | ? | 0.052 | 4.0E-09 |
| rs4665972 |  | rs780094 | 0.862 | Liu JZ | 2015 | Crohn's disease | ? |  | 4.0E-22 |
| rs4665972 |  | rs780094 | 0.862 | Huffman JE | 2015 | Urate levels in obese individuals | C | 0.085 | 2.0E-08 |
| rs4665972 |  | rs780094 | 0.862 | Wood AR | 2014 | Height | T | 0.021 | 6.0E-12 |
| rs4665972 |  | rs780094 | 0.862 | Ram R | 2016 | Hypertriglyceridemia | T | 1.220 | 2.0E-08 |
| rs4665972 |  | rs780094 | 0.862 | Nakayama A | 2016 | Renal underexcretion gout | T | 1.350 | 2.0E-09 |
| rs4665972 |  | rs780094 | 0.862 | Lemaitre RN | 2011 | Plasma omega-3 polyunsaturated fatty acid levels (docosapentaenoic acid) | T | 0.020 | 9.0E-09 |
| rs4665972 |  | rs780094 | 0.862 | He L | 2016 | Age-related disease endophenotypes | ? |  | 8.0E-101 |
| rs4665972 |  | rs780094 | 0.862 | He L | 2016 | Age-related diseases, mortality and associated endophenotypes | ? |  | 4.0E-91 |
| rs4665972 |  | rs780094 | 0.862 | Spracklen CN | 2017 | Triglyceride levels | T | 0.100 | 7.0E-30 |
| rs4665972 |  | rs780094 | 0.862 | Hu Y | 2017 | Gondoic acid (20:1n-9) levels | T | 0.002 | 3.0E-08 |
| rs4665972 |  | rs780094 | 0.862 | Astle WJ | 2016 | Red blood cell count | C | 0.021 | 8.0E-09 |
| rs4665972 |  | rs780094 | 0.862 | Zhao W | 2017 | Type 2 diabetes | C | 0.064 | 2.0E-17 |
| rs4665972 |  | rs780094 | 0.862 | Dupuis J | 2010 | Fasting blood insulin | C | 0.032 | 4.0E-20 |
| rs4665972 |  | rs780094 | 0.862 | Dupuis J | 2010 | Homeostasis model assessment of insulin resistance | C | 0.035 | 3.0E-24 |
| rs4665972 |  | rs780094 | 0.862 | Dupuis J | 2010 | Fasting blood glucose | C | 0.029 | 6.0E-38 |
| rs4665972 |  | rs780094 | 0.862 | Manning AK | 2012 | Fasting blood insulin | T | 0.019 | 3.0E-09 |
| rs4665972 |  | rs780094 | 0.862 | Manning AK | 2012 | Fasting blood glucose | T | 0.032 | 3.0E-24 |
| rs4665972 |  | rs780094 | 0.862 | Manning AK | 2012 | Fasting blood insulin (BMI interaction) | T | 0.001 | 3.0E-10 |
| rs4665972 |  | rs780094 | 0.862 | Manning AK | 2012 | Fasting blood glucose (BMI interaction) | T | 0.001 | 4.0E-24 |
| rs4665972 |  | rs780094 | 0.862 | Kawaguchi T | 2018 | Nonalcoholic fatty liver disease | T | 1.350 | 2.0E-08 |
| rs4665972 |  | rs780093 | 0.856 | Yang Q | 2010 | Urate levels | T | 5.150 | 4.0E-17 |
| rs4665972 |  | rs780093 | 0.856 | Kraja AT | 2011 | Triglycerides-Blood Pressure (TG-BP) | A | 0.180 | 3.0E-10 |
| rs4665972 |  | rs780093 | 0.856 | Kraja AT | 2011 | Waist Circumference - Triglycerides (WC-TG) | A | 0.190 | 2.0E-12 |
| rs4665972 |  | rs780093 | 0.856 | Coviello AD | 2012 | Sex hormone-binding globulin levels | T | 0.032 | 2.0E-16 |
| rs4665972 |  | rs780093 | 0.856 | Coviello AD | 2012 | Sex hormone-binding globulin levels | T | 0.041 | 9.0E-11 |
| rs4665972 |  | rs780093 | 0.856 | Franke A | 2010 | Crohn's disease | T | 1.150 | 5.0E-11 |
| rs4665972 |  | rs780093 | 0.856 | Wu JH | 2013 | Palmitoleic acid (16:1n-7) levels | T | 0.020 | 1.0E-09 |
| rs4665972 |  | rs780093 | 0.856 | Below JE | 2016 | Triglycerides | T | 0.108 | 3.0E-15 |
| rs4665972 |  | rs780093 | 0.856 | KilpelÃƒÂ¤inen TO | 2016 | circulating leptin levels | C | 0.032 | 2.0E-10 |
| rs4665972 |  | rs780093 | 0.856 | KilpelÃƒÂ¤inen TO | 2016 | circulating leptin levels adjusted for BMI | C | 0.024 | 4.0E-10 |
| rs4665972 |  | rs780093 | 0.856 | Spracklen CN | 2017 | Total cholesterol levels | T | 0.052 | 3.0E-47 |
| rs4665972 |  | rs780093 | 0.856 | Hu Y | 2017 | Palmitoleic acid (16:1n-7) levels | T | 0.019 | 4.0E-12 |
| rs4665972 |  | rs780093 | 0.856 | Gorski M | 2017 | Glomerular filtration rate (creatinine) | T | 0.008 | 2.0E-16 |
| rs4665972 |  | rs1260326 | 0.898 | Chambers JC | 2008 | Waist circumference and related phenotypes | ? |  | 4.0E-08 |
| rs4665972 |  | rs1260326 | 0.898 | Sabatti C | 2008 | Metabolic traits | A | 0.090 | 4.0E-10 |
| rs4665972 |  | rs1260326 | 0.898 | Kathiresan S | 2008 | Triglycerides | T | 0.120 | 2.0E-31 |
| rs4665972 |  | rs1260326 | 0.898 | Johansen CT | 2010 | Hypertriglyceridemia | T | 1.750 | 7.0E-09 |
| rs4665972 |  | rs1260326 | 0.898 | Middelberg RP | 2011 | Cardiovascular disease risk factors | T | 0.082 | 2.0E-08 |
| rs4665972 |  | rs1260326 | 0.898 | Chambers JC | 2011 | Liver enzyme levels (gamma-glutamyl transferase) | T | 3.200 | 4.0E-13 |
| rs4665972 |  | rs1260326 | 0.898 | Teslovich TM | 2010 | Triglycerides | T | 8.760 | 6.0E-133 |
| rs4665972 |  | rs1260326 | 0.898 | Teslovich TM | 2010 | Cholesterol, total | T | 1.910 | 7.0E-27 |
| rs4665972 |  | rs1260326 | 0.898 | Dehghan A | 2011 | C-reactive protein levels | T | 0.072 | 5.0E-40 |
| rs4665972 |  | rs1260326 | 0.898 | Kettunen J | 2012 | Metabolite levels | ? | 0.150 | 3.0E-18 |
| rs4665972 |  | rs1260326 | 0.898 | Osman W | 2012 | Non-albumin protein levels | C | 0.080 | 3.0E-09 |
| rs4665972 |  | rs1260326 | 0.898 | Inouye M | 2012 | Metabolite levels | ? |  | 1.0E-12 |
| rs4665972 |  | rs1260326 | 0.898 | Gieger C | 2011 | Platelet count | T | 2.334 | 9.0E-10 |
| rs4665972 |  | rs1260326 | 0.898 | Franceschini N | 2012 | Serum albumin level | T | 0.012 | 3.0E-14 |
| rs4665972 |  | rs1260326 | 0.898 | Franceschini N | 2012 | Serum albumin level | T | 0.027 | 2.0E-08 |
| rs4665972 |  | rs1260326 | 0.898 | Franceschini N | 2012 | Serum albumin level | T | 0.014 | 4.0E-19 |
| rs4665972 |  | rs1260326 | 0.898 | Kottgen A | 2012 | Urate levels | T | 0.074 | 1.0E-44 |
| rs4665972 |  | rs1260326 | 0.898 | Hayes MG | 2013 | Glycemic traits (pregnancy) | T | 0.004 | 6.0E-13 |
| rs4665972 |  | rs1260326 | 0.898 | Hayes MG | 2013 | Glycemic traits (pregnancy) | T | 0.012 | 6.0E-11 |
| rs4665972 |  | rs1260326 | 0.898 | Chasman DI | 2009 | Lipid metabolism phenotypes | ? | 0.075 | 4.0E-32 |
| rs4665972 |  | rs1260326 | 0.898 | Chasman DI | 2009 | Lipid metabolism phenotypes | ? | 0.070 | 1.0E-37 |
| rs4665972 |  | rs1260326 | 0.898 | Chasman DI | 2009 | Lipid metabolism phenotypes | ? | 0.056 | 3.0E-29 |
| rs4665972 |  | rs1260326 | 0.898 | Chasman DI | 2009 | Lipid metabolism phenotypes | ? | 0.052 | 3.0E-35 |
| rs4665972 |  | rs1260326 | 0.898 | Chasman DI | 2009 | Lipid metabolism phenotypes | ? | 0.362 | 4.0E-24 |
| rs4665972 |  | rs1260326 | 0.898 | Chasman DI | 2009 | Lipid metabolism phenotypes | ? | 0.342 | 3.0E-28 |
| rs4665972 |  | rs1260326 | 0.898 | Willer CJ | 2013 | Cholesterol, total | T | 0.051 | 3.0E-42 |
| rs4665972 |  | rs1260326 | 0.898 | Saxena R | 2010 | Two-hour glucose challenge | T | 0.070 | 3.0E-10 |
| rs4665972 |  | rs1260326 | 0.898 | Willer CJ | 2013 | Triglycerides | T | 0.115 | 2.0E-239 |
| rs4665972 |  | rs1260326 | 0.898 | Shin SY | 2014 | Blood metabolite levels | T | 0.044 | 1.0E-77 |
| rs4665972 |  | rs1260326 | 0.898 | Shin SY | 2014 | Blood metabolite levels | T | 0.013 | 6.0E-14 |
| rs4665972 |  | rs1260326 | 0.898 | Shin SY | 2014 | Blood metabolite ratios | T | 0.041 | 3.0E-148 |
| rs4665972 |  | rs1260326 | 0.898 | Kamatani Y | 2010 | Hematological and biochemical traits | C | 0.085 | 4.0E-09 |
| rs4665972 |  | rs1260326 | 0.898 | Kamatani Y | 2010 | Triglycerides | C | 0.101 | 1.0E-11 |
| rs4665972 |  | rs1260326 | 0.898 | Weissglas-Volkov D | 2013 | Hypertriglyceridemia | ? | 1.410 | 2.0E-13 |
| rs4665972 |  | rs1260326 | 0.898 | Korostishevsky M | 2015 | Blood metabolite levels | ? |  | 6.0E-56 |
| rs4665972 |  | rs1260326 | 0.898 | Liu JZ | 2015 | Inflammatory bowel disease | A | 1.080 | 1.0E-14 |
| rs4665972 |  | rs1260326 | 0.898 | Pattaro C | 2016 | Glomerular filtration rate (creatinine) | T | 0.007 | 3.0E-14 |
| rs4665972 |  | rs1260326 | 0.898 | Tin A | 2015 | Plasma lactate levels | T | 0.080 | 4.0E-52 |
| rs4665972 |  | rs1260326 | 0.898 | Tin A | 2015 | Plasma lactate levels | T | 0.080 | 2.0E-47 |
| rs4665972 |  | rs1260326 | 0.898 | Surakka I | 2015 | Cholesterol, total | T | 0.045 | 3.0E-13 |
| rs4665972 |  | rs1260326 | 0.898 | Surakka I | 2015 | Triglycerides | T | 0.123 | 5.0E-88 |
| rs4665972 |  | rs1260326 | 0.898 | Setoh K | 2015 | Serum alpha1-antitrypsin levels | C | 2.050 | 3.0E-16 |
| rs4665972 |  | rs1260326 | 0.898 | Matsuo H | 2015 | Gout | T | 1.360 | 2.0E-12 |
| rs4665972 |  | rs1260326 | 0.898 | Pattaro C | 2016 | Glomerular filtration rate in non diabetics (creatinine) | T | 0.007 | 2.0E-12 |
| rs4665972 |  | rs1260326 | 0.898 | Eppinga RN | 2016 | Resting heart rate | C | 0.275 | 4.0E-16 |
| rs4665972 |  | rs1260326 | 0.898 | Kottgen A | 2010 | Chronic kidney disease | T | 0.010 | 3.0E-14 |
| rs4665972 |  | rs1260326 | 0.898 | Mahajan A | 2016 | Glomerular filtration rate | C | 0.872 | 6.0E-14 |
| rs4665972 |  | rs1260326 | 0.898 | Ligthart S | 2016 | C-reactive protein levels or total cholesterol levels | T |  | 3.0E-63 |
| rs4665972 |  | rs1260326 | 0.898 | Ligthart S | 2016 | C-reactive protein levels or triglyceride levels | T |  | 4.0E-151 |
| rs4665972 |  | rs1260326 | 0.898 | Nakayama A | 2016 | Renal overload gout | T | 1.350 | 5.0E-09 |
| rs4665972 |  | rs1260326 | 0.898 | Nakayama A | 2016 | Gout | T | 1.310 | 7.0E-11 |
| rs4665972 |  | rs1260326 | 0.898 | Kettunen J | 2016 | Metabolite levels | C | 0.100 | 7.0E-26 |
| rs4665972 |  | rs1260326 | 0.898 | de Lange KM | 2017 | Crohn's disease | ? |  | 6.0E-11 |
| rs4665972 |  | rs1260326 | 0.898 | Spracklen CN | 2017 | Triglyceride levels | T | 0.115 | 4.0E-253 |
| rs4665972 |  | rs1260326 | 0.898 | Astle WJ | 2016 | Lymphocyte counts | C | 0.026 | 2.0E-12 |
| rs4665972 |  | rs1260326 | 0.898 | Astle WJ | 2016 | Myeloid white cell count | C | 0.029 | 5.0E-15 |
| rs4665972 |  | rs1260326 | 0.898 | Astle WJ | 2016 | Neutrophil count | C | 0.033 | 1.0E-19 |
| rs4665972 |  | rs1260326 | 0.898 | Astle WJ | 2016 | Sum neutrophil eosinophil counts | C | 0.032 | 2.0E-18 |
| rs4665972 |  | rs1260326 | 0.898 | Astle WJ | 2016 | Sum basophil neutrophil counts | C | 0.033 | 5.0E-19 |
| rs4665972 |  | rs1260326 | 0.898 | Astle WJ | 2016 | Granulocyte count | C | 0.032 | 7.0E-18 |
| rs4665972 |  | rs1260326 | 0.898 | Astle WJ | 2016 | Hematocrit | C | 0.022 | 8.0E-10 |
| rs4665972 |  | rs1260326 | 0.898 | Astle WJ | 2016 | Red cell distribution width | C | 0.025 | 9.0E-12 |
| rs4665972 |  | rs1260326 | 0.898 | Astle WJ | 2016 | White blood cell count | C | 0.034 | 9.0E-21 |
| rs4665972 |  | rs1260326 | 0.898 | Astle WJ | 2016 | High light scatter reticulocyte percentage of red cells | C | 0.035 | 4.0E-22 |
| rs4665972 |  | rs1260326 | 0.898 | Astle WJ | 2016 | Plateletcrit | C | 0.036 | 1.0E-21 |
| rs4665972 |  | rs1260326 | 0.898 | Astle WJ | 2016 | Platelet count | C | 0.039 | 2.0E-25 |
| rs4665972 |  | rs1260326 | 0.898 | Clarke TK | 2017 | Alcohol consumption | T | 0.028 | 1.0E-21 |
| rs4665972 |  | rs1260326 | 0.898 | Clarke TK | 2017 | Alcohol consumption in current drinkers | G | 0.030 | 7.0E-24 |
| rs4665972 |  | rs1260326 | 0.898 | Kawaguchi T | 2018 | Nonalcoholic fatty liver disease | T | 1.380 | 1.0E-09 |
| rs4665972 |  | rs4665972 | 1.000 | Coram MA | 2013 | Triglycerides | T | 0.065 | 1.0E-08 |
| rs4665972 |  | rs4665972 | 1.000 | Astle WJ | 2016 | Reticulocyte fraction of red cells | C | 0.039 | 2.0E-26 |
| rs4665972 |  | rs4665972 | 1.000 | Astle WJ | 2016 | High light scatter reticulocyte count | C | 0.032 | 1.0E-18 |
| rs4665972 |  | rs4665972 | 1.000 | Astle WJ | 2016 | Reticulocyte count | C | 0.034 | 4.0E-20 |
| rs10207567 | *ICA1L* | rs140244541 | 0.807 | Surakka I | 2015 | LDL cholesterol | G | 0.050 | 6.0E-09 |
| rs1047891 | *CPS1* | rs715 | 0.930 | Sabater-Lleal M | 2013 | Fibrinogen | T | 0.009 | 2.0E-11 |
| rs1047891 |  | rs715 | 0.930 | Shin SY | 2014 | Blood metabolite levels | T | 0.076 | 2.0E-147 |
| rs1047891 |  | rs715 | 0.930 | Shin SY | 2014 | Blood metabolite levels | T | 0.091 | 7.0E-58 |
| rs1047891 |  | rs715 | 0.930 | Shin SY | 2014 | Blood metabolite levels | T | 0.033 | 7.0E-35 |
| rs1047891 |  | rs715 | 0.930 | Shin SY | 2014 | Blood metabolite levels | T | 0.045 | 1.0E-24 |
| rs1047891 |  | rs715 | 0.930 | Shin SY | 2014 | Blood metabolite levels | T | 0.022 | 3.0E-21 |
| rs1047891 |  | rs715 | 0.930 | Shin SY | 2014 | Blood metabolite levels | T | 0.022 | 2.0E-19 |
| rs1047891 |  | rs715 | 0.930 | Shin SY | 2014 | Blood metabolite levels | T | 0.036 | 2.0E-16 |
| rs1047891 |  | rs715 | 0.930 | Shin SY | 2014 | Blood metabolite levels | T | 0.022 | 4.0E-16 |
| rs1047891 |  | rs715 | 0.930 | Xie W | 2013 | Metabolite levels | T | 0.610 | 3.0E-50 |
| rs1047891 |  | rs715 | 0.930 | Raffler J | 2015 | Urinary metabolites | C | 0.141 | 8.0E-31 |
| rs1047891 |  | rs715 | 0.930 | Raffler J | 2015 | Urinary metabolites | C | 0.176 | 3.0E-25 |
| rs1047891 |  | rs715 | 0.930 | Hartiala JA | 2016 | Betaine levels in individuals undergoing cardiac evaluation | C | 0.050 | 1.0E-08 |
| rs1047891 |  | rs715 | 0.930 | Draisma HH | 2015 | Amino acid levels | T | 0.031 | 3.0E-11 |
| rs1047891 |  | rs715 | 0.930 | de Vries PS | 2015 | Fibrinogen levels | C | 0.008 | 4.0E-16 |
| rs1047891 |  | rs715 | 0.930 | Mahajan A | 2016 | Glomerular filtration rate | C | 0.876 | 1.0E-11 |
| rs1047891 |  | rs715 | 0.930 | Scerri TS | 2017 | Macular telangiectasia type 2 | ? | 1.923 | 1.0E-15 |
| rs1047891 |  | rs715 | 0.930 | de Vries PS | 2017 | Fibrinogen levels | ? | 0.009 | 9.0E-14 |
| rs1047891 |  | rs715 | 0.930 | de Vries PS | 2017 | Fibrinogen levels | ? | 0.008 | 2.0E-13 |
| rs1047891 |  | rs715 | 0.930 | Astle WJ | 2016 | Eosinophil percentage of white cells | C | 0.024 | 4.0E-10 |
| rs1047891 |  | rs715 | 0.930 | Astle WJ | 2016 | Eosinophil percentage of granulocytes | C | 0.023 | 3.0E-09 |
| rs1047891 |  | rs715 | 0.930 | Astle WJ | 2016 | Plateletcrit | C | 0.024 | 1.0E-09 |
| rs1047891 |  | rs1047891 | 1.000 | van Meurs JB | 2013 | Homocysteine levels | A | 0.086 | 5.0E-27 |
| rs1047891 |  | rs1047891 | 1.000 | Willer CJ | 2013 | HDL cholesterol | A | 0.027 | 9.0E-10 |
| rs1047891 |  | rs1047891 | 1.000 | Danik JS | 2009 | Fibrinogen | A | 4.840 | 9.0E-09 |
| rs1047891 |  | rs1047891 | 1.000 | Lange LA | 2010 | Homocysteine levels | A | 0.050 | 5.0E-09 |
| rs1047891 |  | rs1047891 | 1.000 | Williams SR | 2014 | Plasma homocysteine levels (post-methionine load test) | A | 0.191 | 9.0E-13 |
| rs1047891 |  | rs1047891 | 1.000 | Yu B | 2014 | Serum metabolite levels | A | 0.100 | 4.0E-12 |
| rs1047891 |  | rs1047891 | 1.000 | Pattaro C | 2016 | Chronic kidney disease | A | 1.110 | 8.0E-09 |
| rs1047891 |  | rs1047891 | 1.000 | Pattaro C | 2016 | Glomerular filtration rate (creatinine) | A | 0.011 | 2.0E-23 |
| rs1047891 |  | rs1047891 | 1.000 | Pattaro C | 2016 | Glomerular filtration rate in non diabetics (creatinine) | A | 0.011 | 3.0E-22 |
| rs1047891 |  | rs1047891 | 1.000 | Kottgen A | 2010 | Chronic kidney disease | A | 0.010 | 1.0E-15 |
| rs1047891 |  | rs1047891 | 1.000 | Kettunen J | 2016 | Metabolite levels (small molecules and protein measures) | A | 0.490 | 1.0E-300 |
| rs1047891 |  | rs1047891 | 1.000 | Gorski M | 2017 | Glomerular filtration rate (creatinine) | A | 0.009 | 2.0E-16 |
| rs1047891 |  | rs1047891 | 1.000 | Astle WJ | 2016 | Mean corpuscular volume | A | 0.024 | 1.0E-10 |
| rs1047891 |  | rs1047891 | 1.000 | Astle WJ | 2016 | Mean platelet volume | A | 0.029 | 7.0E-14 |
| rs1047891 |  | rs1047891 | 1.000 | Astle WJ | 2016 | Mean corpuscular hemoglobin | A | 0.024 | 1.0E-10 |
| rs1047891 |  | rs1047891 | 1.000 | Astle WJ | 2016 | Platelet count | A | 0.034 | 2.0E-18 |
| rs10023335 | *SHROOM3* | rs9992101 | 0.999 | Chambers JC | 2010 | Creatinine levels | ? |  | 6.0E-09 |
| rs4865796 | *ARL15* | rs702634 | 0.994 | Mahajan A | 2014 | Type 2 diabetes | A | 1.060 | 7.0E-09 |
| rs4865796 |  | rs702634 | 0.994 | Astle WJ | 2016 | Reticulocyte fraction of red cells | A | 0.026 | 1.0E-11 |
| rs4865796 |  | rs702634 | 0.994 | Astle WJ | 2016 | High light scatter reticulocyte count | A | 0.024 | 5.0E-10 |
| rs4865796 |  | rs702634 | 0.994 | Zhao W | 2017 | Type 2 diabetes | A | 0.050 | 3.0E-09 |
| rs11242113* | *C5orf56* | rs1012793 | 0.998 | de Vries PS | 2017 | Fibrinogen levels | ? | 0.021 | 4.0E-60 |
| rs11242113* |  | rs1012793 | 0.998 | de Vries PS | 2017 | Fibrinogen levels | ? | 0.021 | 1.0E-58 |
| rs11242113* |  | rs2106854 | 0.976 | Sabater-Lleal M | 2013 | Fibrinogen | T | 0.019 | 2.0E-48 |
| rs11242113* |  | rs1016988 | 0.905 | Danik JS | 2009 | Fibrinogen | G | 6.840 | 1.0E-12 |
| rs11242113* |  | rs2057655 | 0.934 | de Vries PS | 2015 | Fibrinogen levels | A | 0.020 | 2.0E-73 |
| rs11242113* |  | rs2522056 | 0.940 | Dehghan A | 2009 | Fibrinogen | A | 0.060 | 1.0E-15 |
| rs11242113* |  | rs2522056 | 0.940 | Astle WJ | 2016 | Lymphocyte counts | A | 0.030 | 2.0E-10 |
| rs1544935* | *KCNK5* | rs1544935 | 1.000 | Nikpay M | 2015 | Myocardial infarction | T | 1.080 | 3.0E-08 |
| rs1544935* |  | rs56336142 | 0.914 | Nikpay M | 2015 | Coronary artery disease | T | 1.070 | 2.0E-08 |
| rs3734692* | *VEGFA* | rs9472138 | 0.865 | Porcu E | 2013 | Thyroid hormone levels | T | 0.079 | 7.0E-16 |
| rs3734692* |  | rs9472138 | 0.865 | Porcu E | 2013 | Thyroid hormone levels | T | 0.090 | 6.0E-12 |
| rs4410790 | *AHR* | rs4410790 | 1.000 | Cornelis MC | 2011 | Caffeine consumption | T | 0.150 | 2.0E-19 |
| rs4410790 |  | rs4410790 | 1.000 | Cornelis MC | 2014 | Coffee consumption (cups per day) | T | 0.100 | 3.0E-17 |
| rs4410790 |  | rs4410790 | 1.000 | Cornelis MC | 2016 | Caffeine metabolism (plasma 1,3,7-trimethylxanthine level) | T | 7.360 | 2.0E-13 |
| rs4410790 |  | rs4410790 | 1.000 | Cornelis MC | 2016 | Caffeine metabolism (plasma 1,7-dimethylxanthine (paraxanthine) to 1,3,7-trimethylxanthine (caffeine) ratio) | T | 8.070 | 7.0E-16 |
| rs4410790 |  | rs6968554 | 0.965 | Shin SY | 2014 | Blood metabolite levels | A | 0.064 | 9.0E-14 |
| rs4410790 |  | rs6968554 | 0.965 | Cornelis MC | 2014 | Coffee consumption | A | 0.200 | 7.0E-15 |
| rs4410790 |  | rs6968554 | 0.965 | Cornelis MC | 2016 | Caffeine metabolism (plasma 1,3,7-trimethylxanthine (caffeine) level) | A | 7.220 | 5.0E-13 |
| rs4410790 |  | rs6968554 | 0.965 | Cornelis MC | 2016 | Caffeine metabolism (plasma 1,7-dimethylxanthine (paraxanthine) to 1,3,7-trimethylxanthine (caffeine) ratio) | A | 8.440 | 3.0E-17 |
| rs4410790 |  | rs6968865 | 0.983 | Sulem P | 2011 | Coffee consumption | T | 0.260 | 2.0E-11 |
| rs2023844 | *HOTTIP* | rs1859168 | 0.999 | Wain LV | 2017 | Diastolic blood pressure | C | 0.464 | 1.0E-08 |
| rs2023844 |  | rs1859168 | 0.999 | Wain LV | 2017 | Diastolic blood pressure | C | 0.436 | 2.0E-20 |
| rs11990607* | *ZBTB10* | rs6998967 | 0.998 | Seldin MF | 2015 | Late-onset myasthenia gravis | G | 1.887 | 9.0E-10 |
| rs28601761 | *TRIB1* | rs28601761 | 1.000 | Astle WJ | 2016 | Mean corpuscular hemoglobin concentration | G | 0.034 | 9.0E-22 |
| rs28601761 |  | rs28601761 | 1.000 | Astle WJ | 2016 | Red blood cell count | G | 0.026 | 7.0E-13 |
| rs28601761 |  | rs28601761 | 1.000 | Astle WJ | 2016 | Mean corpuscular hemoglobin | G | 0.029 | 1.0E-15 |
| rs28601761 |  | rs28601761 | 1.000 | van der Harst P | 2017 | Coronary artery disease | C | 0.060 | 5.0E-20 |
| rs2068888* | *CYP26A1* | rs2068888 | 1.000 | Teslovich TM | 2010 | Triglycerides | A | 2.280 | 2.0E-08 |
| rs2068888* |  | rs2068888 | 1.000 | Willer CJ | 2013 | Triglycerides | A | 0.024 | 2.0E-11 |
| rs2068888* |  | rs2068888 | 1.000 | Spracklen CN | 2017 | Triglyceride levels | A | 0.024 | 3.0E-12 |
| rs2068888* |  | rs2068888 | 1.000 | Astle WJ | 2016 | Plateletcrit | A | 0.024 | 9.0E-11 |
| rs2068888* |  | rs2068888 | 1.000 | Astle WJ | 2016 | Platelet count | A | 0.024 | 9.0E-11 |
| rs3116613* | *DLEU1/BCMS* | rs3116602 | 0.934 | Weedon MN | 2008 | Height | G | 0.040 | 7.0E-09 |
| rs3116613* |  | rs3118910 | 0.934 | Shungin D | 2015 | Hip circumference | T | 0.041 | 3.0E-12 |
| rs3116613* |  | rs3118914 | 0.933 | Soranzo N | 2009 | Height | ? | 0.080 | 4.0E-10 |
| rs4899263 | *ZFP36L1* | rs10142466 | 0.801 | Liu JZ | 2015 | Inflammatory bowel disease | ? |  | 1.0E-08 |
| rs60476496 | *SPATA5L1* | rs61524473 | 0.994 | Kettunen J | 2016 | Metabolite levels (small molecules and protein measures) | C | 0.090 | 1.0E-15 |
| rs60476496 |  | rs1153858 | 0.969 | Kleber ME | 2013 | Homoarginine levels | C | 0.271 | 1.0E-45 |
| rs2472297 | *CYP1A1/CYP1A2* | rs2472297 | 1.000 | Sulem P | 2011 | Coffee consumption | T | 0.310 | 5.0E-14 |
| rs2472297 |  | rs2472297 | 1.000 | Cornelis MC | 2014 | Coffee consumption (cups per day) | T | 0.140 | 2.0E-24 |
| rs2472297 |  | rs2472297 | 1.000 | Cornelis MC | 2016 | Caffeine metabolism (plasma 1,3,7-trimethylxanthine (caffeine) level) | T | 9.340 | 1.0E-20 |
| rs2472297 |  | rs2472297 | 1.000 | Cornelis MC | 2016 | Caffeine metabolism (plasma 1,7-dimethylxanthine (paraxanthine) to 1,3,7-trimethylxanthine (caffeine) ratio) | T | 9.580 | 9.0E-22 |
| rs784257 | *TCF4/LINC01415* | rs613872 | 0.843 | Baratz KH | 2010 | Fuchs's corneal dystrophy | G | 5.470 | 1.0E-18 |
| rs784257 |  | rs784257 | 1.000 | Afshari NA | 2017 | Fuchs's corneal dystrophy | ? | 4.940 | 3.0E-200 |
| rs56254331* | *CCDC97* | rs73045269 | 0.992 | van der Harst P | 2017 | Coronary artery disease | T | 0.070 | 1.0E-14 |

**Supplementary Table 6.** Results from COLOC analysis for ACR associated variants previously known to be associated with other traits. Results based on beta and beta variance estimates are provided alongside P-value based results where available from the NHGRI-EBI GWAS catalog. NA given when data unavailable for beta and beta-variance based COLOC analysis. Novel indicates if lead SNP has not previously been reported to be associated with ACR. * FG=fasting glucose; FI=fasting insulin; T2D=type 2 diabetes; TC=total cholesterol; TRIG=triglycerides; LDL=low-density lipoprotein; HDL=high-density lipoprotein; CAD=coronary artery disease; DBP=diastolic blood pressure. †Probability both traits are associated at the locus with different causal variants. ‡Probability both traits are associated at the locus with the same causal variant.

|  |  |  |  |  |  |  | **Beta and Beta Variance Analysis** | | **P-value Analysis** | |
| --- | --- | --- | --- | --- | --- | --- | --- | --- | --- | --- |
| **Locus** | **ACR SNP** | **Novel** | **Chromosome** | **Position (HG19)** | **TRAIT*** | **STUDY** | **Prob(Diff)**† | **Prob(Same)**‡ | **Prob(Diff)**† | **Prob(Same)**‡ |
| *SNX17* | rs4665972 | No | 2 | 27598097 | FG | Manning et al (2012) | 7.2E-04 | 1.0E+00 | 6.5E-03 | 9.9E-01 |
| *SNX17* | rs4665972 | No | 2 | 27598097 | FI | Manning et al (2012) | 9.5E-04 | 1.0E+00 | 2.5E-03 | 1.0E+00 |
| *SNX17* | rs4665972 | No | 2 | 27598097 | T2D | Zhao et al (2017) | 2.9E-01 | 7.1E-01 | 3.1E-01 | 6.9E-01 |
| *SNX17* | rs4665972 | No | 2 | 27598097 | TC | Teslovich et al (2010) | NA | NA | 1.5E-04 | 1.0E+00 |
| *SNX17* | rs4665972 | No | 2 | 27598097 | TC | Willer et al (2013) | 2.8E-03 | 1.0E+00 | 1.3E-03 | 1.0E+00 |
| *SNX17* | rs4665972 | No | 2 | 27598097 | TC | Surakka et al (2015) | 9.1E-03 | 9.9E-01 | 9.3E-03 | 9.9E-01 |
| *SNX17* | rs4665972 | No | 2 | 27598097 | TRIG | Teslovich et al (2010) | NA | NA | 9.9E-01 | 8.2E-03 |
| *SNX17* | rs4665972 | No | 2 | 27598097 | TRIG | Willer et al (2013) | 1.4E-04 | 1.0E+00 | 1.3E-04 | 1.0E+00 |
| *ICA1L* | rs10207567 | No | 2 | 203714973 | LDL | Surakka et al (2015) | 2.0E-01 | 8.0E-01 | 2.0E-01 | 8.0E-01 |
| *CPS1* | rs1047891 | No | 2 | 211540507 | HDL | Willer et al (2013) | 2.5E-06 | 1.0E+00 | 4.2E-06 | 1.0E+00 |
| *ARL15* | rs4865796 | No | 5 | 53272664 | T2D | Zhao et al (2017) | 3.3E-03 | 1.0E+00 | 3.7E-03 | 1.0E+00 |
| *KCNK5* | rs1544935 | Yes | 6 | 39124448 | CAD | Nikpay et al (2015) | 1.6E-02 | 9.8E-01 | 1.6E-02 | 9.8E-01 |
| *HOTTIP* | rs2023844 | No | 7 | 27243238 | DBP | Wain et al (2017) | 8.9E-03 | 9.9E-01 | 9.9E-03 | 9.9E-01 |
| *CYP26A1* | rs2068888 | Yes | 10 | 94839642 | TRIG | Willer et al (2013) | 5.3E-04 | 1.0E+00 | 5.1E-04 | 1.0E+00 |

**Supplementary Table 7. Results of gene-set enrichment derived using MAGMA as implemented in FUMA. Only results with multiple testing corrected P < 0.05 are presented.**

| **Gene Set** | **N genes** | **Beta** | **Beta STD** | **SE** | **P** | **P_bon_** |
| --- | --- | --- | --- | --- | --- | --- |
| GO_bp:go_female_genitalia_development | 15 | 1.17 | 0.0334 | 0.242 | 6.63E-07 | 7.22E-03 |
| Curated_gene_sets:nikolsky_breast_cancer_7p15_amplicon | 11 | 2.98 | 0.0728 | 0.624 | 8.91E-07 | 9.70E-03 |
| GO_bp:go_embryonic_digestive_tract_development | 33 | 0.94 | 0.0396 | 0.202 | 1.67E-06 | 1.82E-02 |
| GO_bp:go_lipoprotein_transport | 12 | 1.63 | 0.0415 | 0.354 | 2.21E-06 | 2.40E-02 |
| GO_bp:go_lipoprotein_localization | 12 | 1.63 | 0.0415 | 0.354 | 2.21E-06 | 2.40E-02 |

**Supplementary Table 8.** UK Biobank individuals classified based on their combination of *CUBN* genotypes for the three SNPs identified as association with ACR at this locus. Percentages of individuals with disease status respective to genotype group for all individuals and those above clinical threshold for albuminuria are shown in brackets. *Standard deviation. †Type 2 diabetes. ‡Chronic kidney disease. §Coronary artery disease. ||Hypertension.

| **Genotypes of SNPs at CUBN locus** | | |  | | | | | | | | **Individuals above clinical threshold (>3 mg/mmol)** | | | | |
| --- | --- | --- | --- | --- | --- | --- | --- | --- | --- | --- | --- | --- | --- | --- | --- |
| **rs45551835**  **(1.5%)** | **rs45619139**  **(10%)** | **rs141640975**  **(0.25%)** | **N** | **Mean**  **ACR (SD^*^)** | **N**  **T2D**  **cases**^†^ | **N**  **CKD**  **cases**^‡^ | **N**  **CAD**  **cases**^§^ | **N**  **Hyper.**  **cases^\|\|^** | **N**  **Stroke**  **cases** | **N**  **>3**  **mg/mmol** | **N**  **T2D**  **cases**^†^ | **N**  **CKD**  **cases**^‡^ | **N**  **CAD**  **cases**^§^ | **N**  **Hyper.**  **cases^\|\|^** | **N**  **Stroke**  **cases** |
| 0 (G-G) | 0 (C-C) | 0 (G-G) | 349,958 | 1.62 (2.41) | 10,846  (3.1) | 3,492  (1.0) | 28,948  (8.3) | 187,169  (53.5) | 6,799  (1.9) | 31,582  (9.0) | 1,791  (5.7) | 852  (2.7) | 3,228  (10.2) | 19,688  (62.3) | 903  (2.9) |
| 0 (G-G) | 0 (C-C) | 1 (G-A) | 2,003 | 2.42 (3.78) | 62  (3.1) | 12  (0.6) | 173  (8.6) | 1,072  (53.5) | 44  (2.2) | 344  (17.2) | 24  (7.0) | 5  (1.5) | 42  (12.2) | 234  (68.0) | 13  (3.8) |
| 0 (G-G) | 0 (C-C) | 2 (A-A) | 7 | 15.94 (5.04) | 0  (0) | 0  (0) | 0  (0) | 1  (14.3) | 0  (0) | 7  (100.0) | 0  (0) | 0  (0) | 0  (0) | 1  (14.3) | 0  (0) |
| 0 (G-G) | 1 (C-G) | 0 (G-G) | 69,109 | 1.66 (2.49) | 2,191  (3.2) | 682  (1.0) | 5,684  (8.2) | 36,270  (52.5) | 1,422  (2.1) | 6,572  (9.5) | 430  (6.5) | 183  (2.8) | 721  (11.0) | 4,009  (61.0) | 223  (3.4) |
| 0 (G-G) | 1 (C-G) | 1 (G-A) | 186 | 3.47 (4.24) | 3  (1.6) | 2  (1.1) | 17  (9.1) | 105  (56.5) | 2  (1.1) | 66  (35.5) | 1  (1.5) | 2  (3.0) | 5  (7.6) | 47  (71.2) | 1  (1.5) |
| 0 (G-G) | 2 (G-G) | 0 (G-G) | 3,403 | 1.74 (2.81) | 101  (3.0) | 40  (1.2) | 258  (7.6) | 1,785  (52.4) | 73  (2.1) | 349  (10.3) | 22  (6.3) | 14  (4.0) | 40  (11.5) | 216  (61.9) | 13  (3.7) |
| 1 (G-A) | 0 (C-C) | 0 (G-G) | 839 | 1.78 (2.97) | 26  (3.1) | 13  (1.5) | 68  (8.1) | 444  (52.9) | 13  (1.5) | 92  (11.0) | 7  (7.6) | 2  (2.2) | 6  (6.5) | 59  (64.1) | 2  (2.2) |
| 1 (G-A) | 1 (C-G) | 0 (G-G) | 10,423 | 1.92 (3.05) | 334  (3.2) | 91  (0.9) | 869  (8.3) | 57,48  (55.1) | 201  (1.9) | 1,246  (12.0) | 90  (7.2) | 37  (3.0) | 161  (12.9) | 870  (69.8) | 41  (3.3) |
| 1 (G-A) | 1 (C-G) | 1 (G-A) | 25 | 7.69 (5.12) | 0  (0) | 0  (0) | 1  (4.0) | 10  (40.0) | 1  (4.0) | 21  (84.0) | 0  (0) | 0  (0) | 0  (0) | 8  (38.1) | 1  (4.8) |
| 1 (G-A) | 2 (G-G) | 0 (G-G) | 986 | 2.07 (2.43) | 37  (3.8) | 11  (1.1) | 82  (8.3) | 534  (54.2) | 23  (2.3) | 174  (17.6) | 16  (9.2) | 3  (1.7) | 17  (9.8) | 112  (64.4) | 8  (4.6) |
| 2 (A-A) | 1 (C-G) | 0 (G-G) | 12 | 3.21 (3.62) | 0  (0) | 0  (0) | 3  (25.0) | 9  (75.0) | 0  (0) | 4  (33.3) | 0  (0) | 0  (0) | 1  (25.0) | 3  (75.0) | 0  (0) |
| 2 (A-A) | 2 (G-G) | 0 (G-G) | 76 | 3.86 (3.68) | 3  (3.9) | 2  (2.6) | 13  (17.1) | 49  (64.5) | 3  (3.9) | 33  (43.4) | 3  (9.1) | 0  (0) | 8  (24.2) | 28  (84.8) | 2  (6.1) |

**Supplementary Table 9**. Effect of associations in the *CUBN* locus with ACR in people with and without diabetes in the UK Biobank. Univariable results when each SNP is entered individually and multivariable results when all three SNPs are entered in the same regression model. *Effect allele / other allele. †Effect allele frequency. ‡Standard error.

|  |  |  |  | **Individuals with diabetes** | | | **Individuals without diabetes** | | |
| --- | --- | --- | --- | --- | --- | --- | --- | --- | --- |
| **SNP** | **EA/OA^*^** | **EA Freq.**^†^ | **Model** | **Beta** | **SE**^‡^ | **P-value** | **Beta** | **SE**^‡^ | **P-value** |
| rs141640975 | A/G | 0.003 | Univariable | 0.781 | 0.107 | 2.6E-13 | 0.449 | 0.022 | 1.3E-89 |
|  |  |  | Multivariable | 0.800 | 0.106 | 5.9E-14 | 0.456 | 0.022 | 2.8E-92 |
| rs45551835 | A/G | 0.014 | Univariable | 0.358 | 0.044 | 3.8E-16 | 0.179 | 0.010 | 1.4E-78 |
|  |  |  | Multivariable | 0.296 | 0.046 | 2.0E-10 | 0.149 | 0.010 | 1.3E-49 |
| rs45619139 | G/C | 0.101 | Univariable | 0.115 | 0.017 | 4.7E-11 | 0.055 | 0.004 | 3.7E-49 |
|  |  |  | Multivariable | 0.078 | 0.018 | 2.2E-05 | 0.037 | 0.004 | 3.9E-21 |

**Supplementary Table 10.** Results of interaction term for 62 ACR associated SNPs with diabetes status. Previously reported SNPs by Teumer *et al.* (2016) also provided. *Effect allele. †Standard error.

| **Locus** | **SNP** | **EA^*^** | **Beta** | **SE**^†^ | **P-value** |
| --- | --- | --- | --- | --- | --- |
| *CUBN* | rs45551835 | A | 0.215 | 0.043 | 5.57E-07 |
| *CUBN* | rs45619139 | G | 0.075 | 0.017 | 1.18E-05 |
| *CUBN* | rs141640975 | A | 0.426 | 0.104 | 4.26E-05 |
| *CPS1* | rs1047891 | A | 0.033 | 0.011 | 3.32E-03 |
| *C10orf11* | rs7898462 | G | 0.036 | 0.012 | 3.43E-03 |
| *SHROOM3* | rs10023335 | C | -0.028 | 0.010 | 7.68E-03 |
| *FGR1* | rs189107782 | T | 0.319 | 0.129 | 1.32E-02 |
| *SNX17* | rs4665972 | C | -0.027 | 0.011 | 1.32E-02 |
| *MYL3* | rs6768627 | T | 0.041 | 0.021 | 4.84E-02 |
| *SPATA5L1* | rs60476496 | T | -0.021 | 0.012 | 7.26E-02 |
| *ADO* | rs10995311 | G | -0.019 | 0.010 | 7.38E-02 |
| *SPHKAP* | rs35924503 | C | 0.280 | 0.159 | 7.77E-02 |
| *CCT2* | rs2601006 | C | -0.019 | 0.011 | 8.12E-02 |
| *KCNK5* | rs1544935 | G | -0.020 | 0.013 | 1.12E-01 |
| *MLLT10* | rs6482189 | A | -0.017 | 0.011 | 1.21E-01 |
| *MSTO1* | rs35202981 | G | -0.023 | 0.015 | 1.25E-01 |
| *DPY19L2P3/WIPF3* | rs9638860 | T | 0.018 | 0.012 | 1.36E-01 |
| *CWC27* | rs7731168 | C | 0.018 | 0.012 | 1.44E-01 |
| *FBXL20* | rs2338796 | G | -0.016 | 0.011 | 1.45E-01 |
| *CYP1A1/CYP1A2* | rs2472297 | T | 0.016 | 0.012 | 1.62E-01 |
| *AUTS2* | rs35692677 | A | -0.018 | 0.013 | 1.70E-01 |
| *NR3C2* | rs6535594 | A | 0.014 | 0.010 | 1.70E-01 |
| *ZBTB46* | rs11697610 | G | -0.015 | 0.011 | 1.73E-01 |
| *ICA1L* | rs10207567 | C | 0.018 | 0.013 | 1.80E-01 |
| *EDEM3* | rs78444298 | A | -0.049 | 0.037 | 1.86E-01 |
| *OAF* | rs12790943 | T | 0.013 | 0.010 | 2.12E-01 |
| *GPD2* | rs111688960 | A | 0.056 | 0.046 | 2.25E-01 |
| *CCDC97* | rs56254331 | C | -0.016 | 0.014 | 2.36E-01 |
| *NUMA1* | rs7115200 | G | 0.012 | 0.010 | 2.44E-01 |
| *CYP2A7* | rs79600176 | T | 0.042 | 0.037 | 2.55E-01 |
| *FUT1* | rs838142 | G | 0.013 | 0.012 | 2.67E-01 |
| *NMU* | rs3805382 | G | -0.012 | 0.011 | 2.80E-01 |
| *ARL15* | rs4865796 | A | -0.010 | 0.011 | 3.69E-01 |
| *FOXD2* | rs1337526 | G | 0.012 | 0.013 | 3.75E-01 |
| *DPM3/KRTCAP2* | rs34257409 | T | 0.009 | 0.010 | 3.93E-01 |
| *BAHCC1* | rs35572189 | A | -0.009 | 0.011 | 4.08E-01 |
| *CYP26A1* | rs2068888 | A | -0.008 | 0.010 | 4.17E-01 |
| *FOXD2* | rs6676159 | T | -0.009 | 0.011 | 4.21E-01 |
| *NYAP2* | rs183131780 | T | 0.090 | 0.119 | 4.51E-01 |
| *COL4A4* | rs35483183 | A | 0.011 | 0.016 | 4.96E-01 |
| *TYRO3* | rs28844285 | T | -0.007 | 0.011 | 5.17E-01 |
| *TRIB1* | rs28601761 | G | -0.007 | 0.011 | 5.27E-01 |
| *PHC2/ZSCAN20* | rs12032996 | A | -0.008 | 0.014 | 5.51E-01 |
| *FAT1* | rs62342738 | C | 0.008 | 0.013 | 5.59E-01 |
| *SBF2* | rs11042685 | C | 0.005 | 0.010 | 6.16E-01 |
| *WDR81* | rs550628400 | G | -0.031 | 0.069 | 6.51E-01 |
| *DLEU1/BCMS* | rs3116613 | G | -0.005 | 0.013 | 6.97E-01 |
| *C5orf56* | rs11242113 | A | -0.005 | 0.013 | 7.05E-01 |
| *HOTTIP* | rs2023844 | A | 0.007 | 0.020 | 7.17E-01 |
| *NAV3* | rs10860332 | A | -0.004 | 0.011 | 7.17E-01 |
| *PARTICL* | rs12714144 | T | 0.005 | 0.016 | 7.43E-01 |
| *PRKCI* | rs112607182 | T | 0.006 | 0.020 | 7.61E-01 |
| *PRRC2C* | rs12727104 | A | -0.005 | 0.018 | 7.66E-01 |
| *TCF4/LINC01415* | rs784257 | C | -0.004 | 0.013 | 7.72E-01 |
| *VEGFA* | rs3734692 | T | 0.002 | 0.011 | 8.45E-01 |
| *FGR1* | rs4109437 | A | -0.005 | 0.027 | 8.46E-01 |
| *ZFP36L1* | rs4899263 | A | -0.002 | 0.010 | 8.53E-01 |
| *ARL15* | rs31226 | C | -0.002 | 0.011 | 8.75E-01 |
| *USP3* | rs146311723 | C | 0.002 | 0.014 | 8.93E-01 |
| *AHR* | rs4410790 | C | -0.001 | 0.011 | 9.44E-01 |
| *ZBTB10* | rs11990607 | G | -0.001 | 0.014 | 9.49E-01 |
| *AK5* | rs11162351 | C | 0.000 | 0.011 | 9.85E-01 |
| *Genetic Risk Score* | | | | | |
| 62-SNP Risk score | | | 0.026 | 0.003 | 1.24E-15 |
| 59-SNP Risk score (SNPs in *CUBN* locus removed) | | | 0.015 | 0.003 | 1.71E-06 |
| *Previously reported SNPS* | | | | | |
| *HS6ST1* | rs13427836 | T | -0.005 | 0.015 | 7.59E-01 |
| *CUBN* | rs1801239 | T | 0.068 | 0.017 | 5.36E-05 |
| *RAB38* | rs649529 | T | -0.020 | 0.010 | 5.68E-02 |

**Supplementary Figure 1.** The distribution of ACR among individuals analysed in the UK Biobank, split by sex and diabetes status.

**
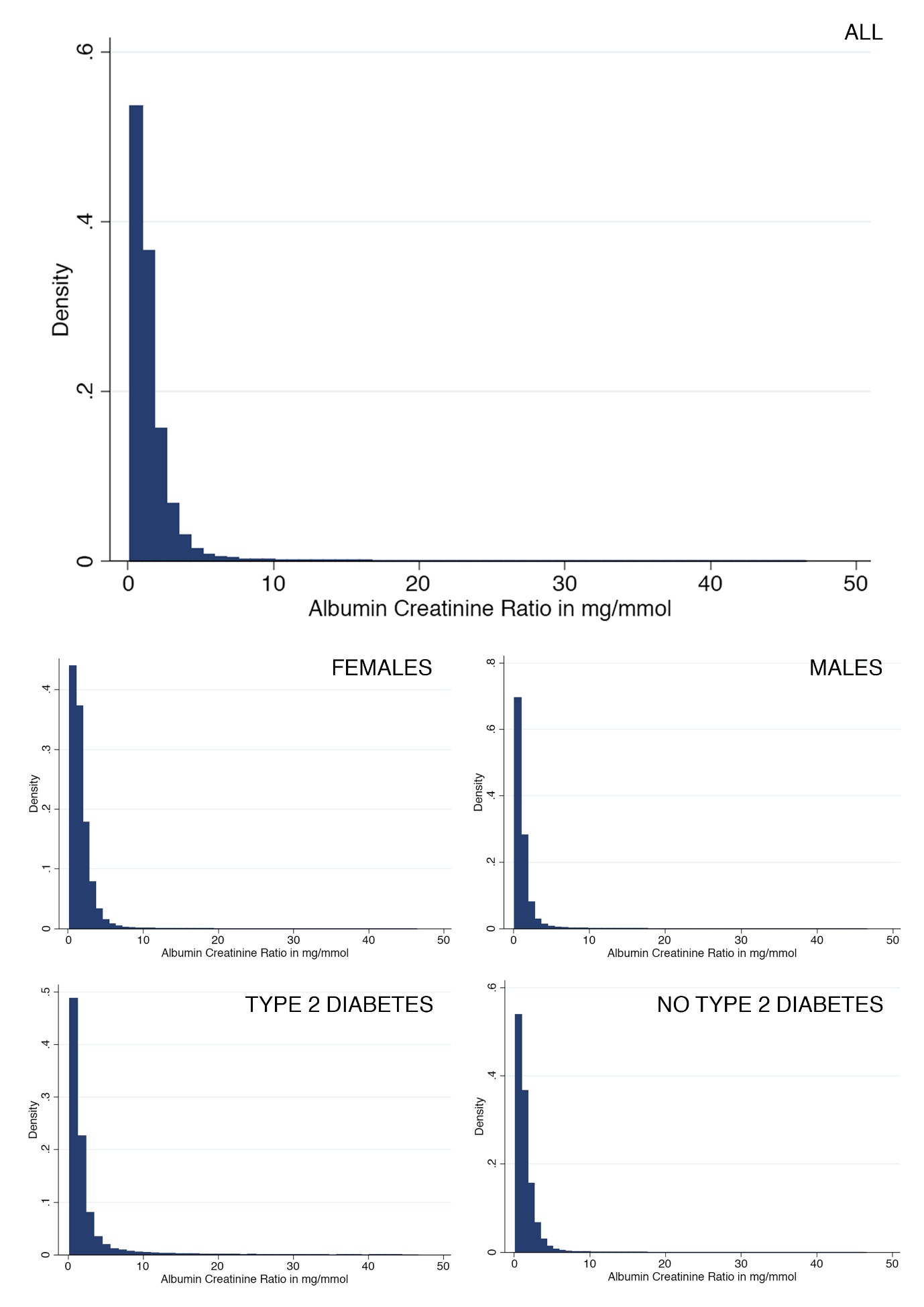
**

**Supplementary Figure 2.** Manhattan plot showing the results obtained from performing a genome-wide association study of ACR in the UK Biobank using BOLT-LMM. The black horizontal line represents P=5x10^-8^. The nearest gene at each locus with evidence of an association at P < 5×10-8 is provided under the corresponding chromosome label.

**Supplementary Figure 3.** Quantile-quantile plot of association statistics from UK Biobank GWAS of ACR. λ inflation factor = 1.20; LD-score intercept = 1.03.


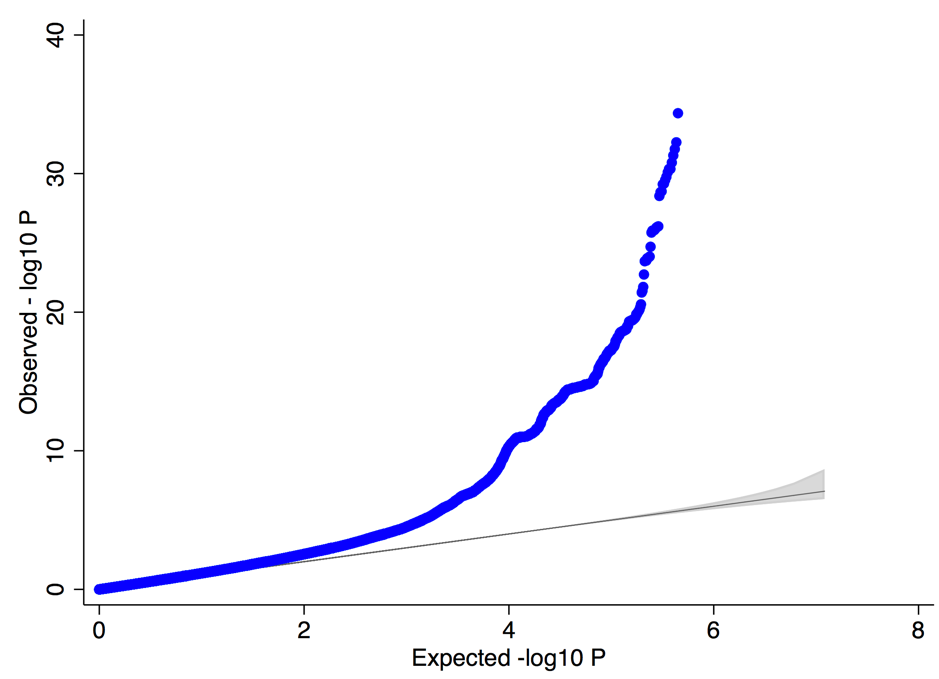


**Supplementary Figure 4**. Results of MAGMA tissue enrichment analysis. No tissue enrichment of gene expression observed for genes in associated loci at Bonferroni P<0.05 after accounting for 53 tissue types.


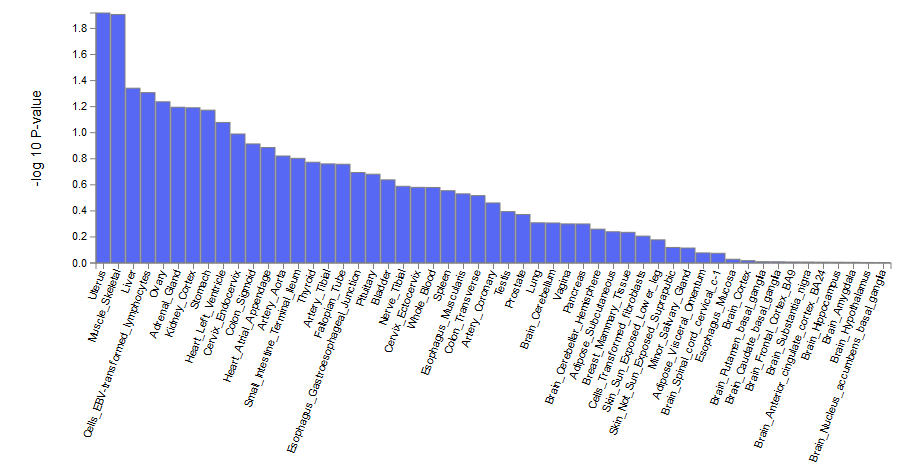


**Supplementary Figure 5.** Histogram of the –log10 p values obtained from 1000 diabetes-interaction analyses of ACR and a) rs141640975, b) rs45551835, c) rs45619139, d) weighted GRS for ACR with all 62 SNPs, and e) weighted GRS for ACR with the 3 SNPs at the CUBN locus removed, when we selected groups of individuals to have the same means and standard deviations of ACR as individuals with and without diagnosis of diabetes, but who were randomised to diabetes status. Red vertical lines represent the observed P-value in UK Biobank for the respective SNP.

**
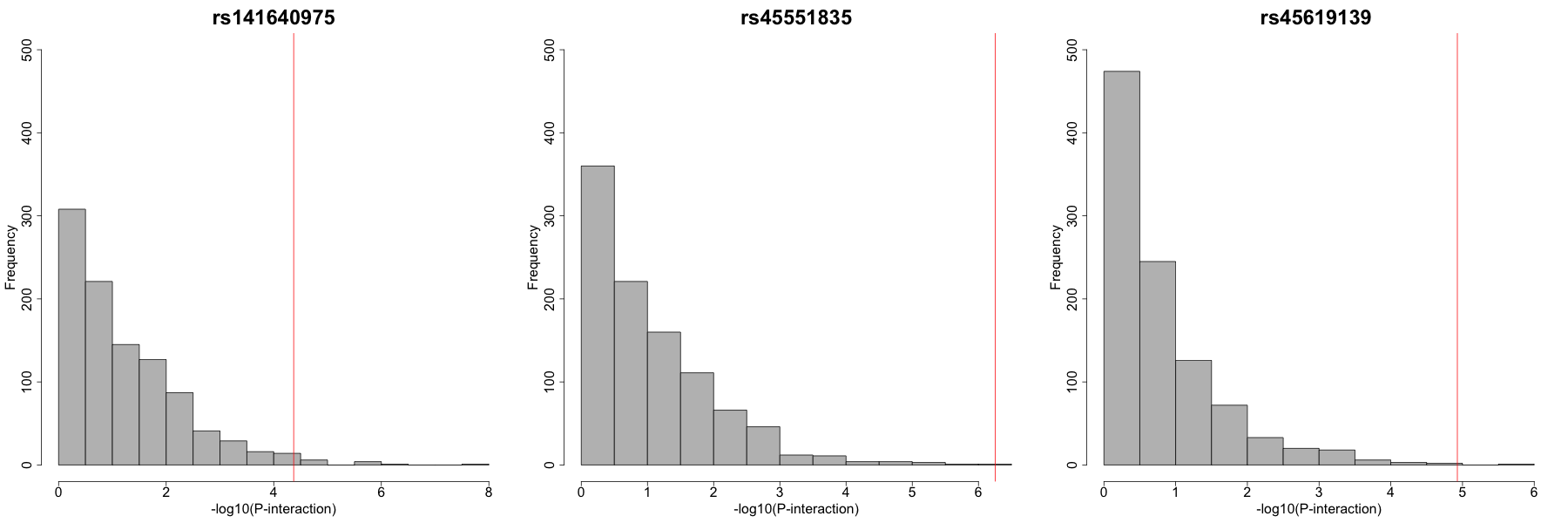
**


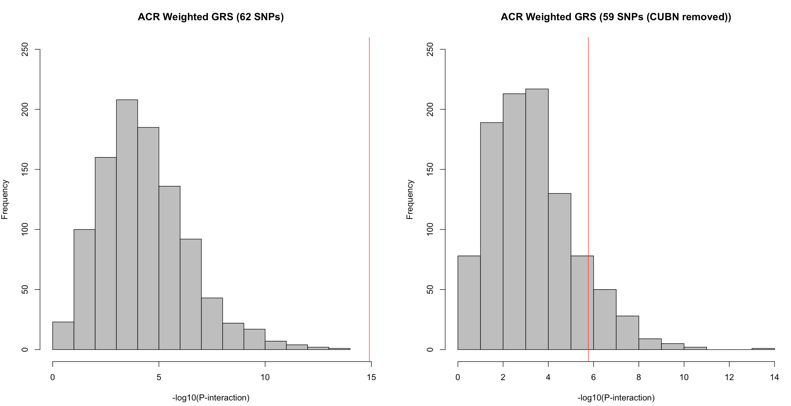

Supplement: 20190927_R2_Supplementary_material_clean_ddz243 [file 20190927_r2_supplementary_material_clean_ddz243.docx]
